# Osborne fractionation of pomegranate seed protein: Structural characteristics, processing properties, proteomic profiles and nutritional value

**DOI:** 10.1016/j.fochx.2026.104551

**Published:** 2026-10-03

**Authors:** Lin Yuan, Yu Qiu, Jiayan Yang, Qianyang Fang, Qianlin Deng, Jingyang Hong, Mengmeng Zhao, Min Zhu

**Affiliations:** a College of Smart Agriculture (Research Institute), Xinjiang University, Urumqi 830046, China; b Xinjiang Silk Research Institute(Fruit and Vegetable Processing), Urumqi 830046, China

**Keywords:** Pomegranate seed protein, Structural characteristics, Nutritional quality, Proteomic analysis

## Abstract

Pomegranate seeds constitute over 50% of juice-processing pomace and represent an underutilized sustainable plant protein source. This study compared albumin (ALB), globulin (GLO), prolamin (PRO), glutelin (GLU), and crude protein (CP) fractions of pomegranate seed protein. ALB exhibited the highest solubility (24.32%) and essential amino acid index (15.24%) with the lowest trypsin inhibition rate (5.08%), indicating superior hydration and nutritional potential. PRO showed the highest surface hydrophobicity (472.62), strongest oil-holding capacity and emulsion stability, the highest intestinal digestibility (21.18%), and the lowest phytic acid content (1.03 μmol/g). GLU exhibited the highest zeta potential (−30.14 mV), thermal stability (Td 163.2 °C; ΔH 201.65 J/g), and foaming performance. Correlation analysis linked secondary structure and surface hydrophobicity with thermal stability, oil binding, emulsification, and digestibility. Data-independent acquisition mass spectrometry revealed fraction-specific enrichment of storage, lipid-transfer-related, and metabolic proteins. These findings demonstrate structure–function differentiation among PSP fractions and support their targeted food applications.

## Highlights

• Pomegranate seed protein fractions show distinct structure–function relationships.
• ALB, PRO, and GLU show distinct processing properties and nutritional value.
• DIA-MS reveals differential proteins driving fraction-specific functionality.

## 1 Introduction

Global food and nutritional insecurity, driven by population growth and climate change, has increased the demand for sustainable and accessible protein resources (Lumsden et al., 2024; Shah et al., 2024). Compared with animal proteins, plant proteins generally offer advantages in terms of production cost, environmental sustainability, and compatibility with health-oriented dietary patterns (Gil et al., 2024). Beyond conventional plant protein materials, such as legumes, underutilized food-processing by-products with valorization potential have gradually emerged as promising sources of novel plant proteins and have attracted increasing research interest (Nirmal et al., 2025). However, most studies on plant proteins have focused mainly on crude protein extracts, whereas plant proteins are generally composed of multiple fractions, including albumins, globulins, prolamins, and glutelins (Oluwajuyitan & Aluko, 2025). These fractions might differ markedly in terms of molecular structure, surface properties, nutritional quality, and digestive behavior.

Pomegranate seed protein (PSP) has garnered attention as a potential plant protein resource. Pomegranate seeds are major by-products of juice processing, accounting for more than 50% of the total pomace, and they contain approximately 20–30% protein. PSP is characterized by a complete amino acid profile and a relatively high proportion of essential amino acids. These nutritional characteristics indicate its potential for high-value food application. However, PSP remains insufficiently utilized despite its nutritional value and availability as a processing by-product (Kandylis & Kokkinomagoulos, 2020; Rahimipanah et al., 2022).

This underutilization might be partly attributed to the limited understanding of the composition-dependent properties of PSP. In particular, the systematic separation and comparative evaluation of individual protein fractions remain insufficient. Osborne sequential fractionation provided an effective strategy for separating plant proteins into albumins, globulins, prolamins, and glutelins based on their solubility differences (Chen et al., 2025). During fractionation, parameters such as solvent polarity, pH, and ionic strength can regulate intra and intermolecular interactions, including hydrogen bonding, hydrophobic interactions, and the formation of disulfide linkages. Such structural remodeling might induce differential changes in the secondary conformation, surface hydrophobicity, and aggregation state. These changes further contribute to the differences in the functional properties and nutritional value of PSP fractions (Rasheed et al., 2020). The importance of protein composition in determining functionality is well established in conventional plant protein systems. For example, soybean protein, one of the most extensively studied and commercially utilized plant proteins, is dominated by the storage globulins β-conglycinin (7S) and glycinin (11S), and variation in their relative abundance and structural characteristics among soybean cultivars has been associated with differences in thermal stability, secondary structure, emulsification, and other functional properties (Feyzi & Ismail, 2026; Ma, Li, et al., 2025). These findings demonstrate that the functional behavior of plant proteins is strongly dependent on their specific protein composition rather than being determined solely by their general botanical origin or protein class. Although structure–function relationships have been investigated for several crop proteins, their application to PSP remains largely unexplored (Guzmán-Lorite et al., 2022). Comparable fraction-level information for PSP remains scarce, and it is still unclear whether its individual protein fractions exhibit compositional, functional, and nutritional characteristics distinct from those of conventional plant protein systems.

Therefore, this study aimed to systematically clarify the structural characteristics, functional properties, nutritional quality, and digestive behavior of PSP and its fractions, with particular emphasis on identifying the fraction-specific characteristics of PSP in the context of established plant protein systems. PSP was prepared using alkaline extraction–isoelectric precipitation and subsequently separated into albumin, globulin, prolamin, and glutelin fractions using Osborne sequential fractionation, enabling an integrated comparison of their structural, functional, nutritional, digestive, and proteomic characteristics. The molecular profiles, structural conformations, colloidal stability, and thermal behavior of these protein samples were comprehensively analyzed by SDS-PAGE, particle size analysis, zeta potential measurement, Fourier-transform infrared spectroscopy (FTIR), circular dichroism spectroscopy (CD), and differential scanning calorimetry (DSC). Furthermore, the nutritional quality of each sample was evaluated using amino acid scoring indices, in vitro protein digestibility (IVPD), and representative antinutritional factors. Proteomics was employed to identify fraction-specific protein compositions and provide a molecular basis for the differences in structure, function, nutritional value, and digestive characteristics among PSP fractions. These findings provide a theoretical basis for the targeted development of PSP and its fractions as sustainable plant protein ingredients and their high-value utilization in food systems.

## 2 Materials and methods

### 2.1 Materials

Pomegranate seeds were obtained from the juice-processing residues of hard seeded sour pomegranates (*Punica granatum* L.) grown in Artux, Xinjiang, China. The fruits were washed and mechanically pressed for juice extraction, and the seeds were recovered from the resulting juice-processing residues. The recovered seeds were washed with water to remove adhering pulp and juice residues, air-dried at ambient temperature (25 ± 5 °C) to constant weight, and stored at 4 °C until use. Before protein extraction, the dried seeds were ground into powder and defatted with petroleum ether. The protein molecular weight marker (10–180 kDa), 8-anilino-1-naphthalenesulfonic acid ammonium salt (ANS), pepsin, and trypsin were purchased from Sinopharm Chemical Reagent Co., Ltd. (Shanghai, China). All reagents were of analytical grade, unless otherwise stated.

### 2.2 PSP extraction and Osborne sequential fractionation

PSP was prepared from defatted pomegranate seed flour by alkaline solubilization followed by isoelectric precipitation, as described by Oroumei et al. (2024), with slight modifications. Four PSP fractions were obtained by sequential Osborne fractionation, following the method described by Ma, Mondor, et al. (2025), with appropriate modifications. Briefly, defatted flour was sequentially extracted with 10 mM Tris-HCl buffer (pH 7.0), 0.5 M NaCl in 10 mM Tris-HCl buffer (pH 7.0), 60% (*v*/v) ethanol containing 5 mM ascorbic acid, and 0.1 M NaOH containing 5 mM ascorbic acid to obtain ALB, GLO, PRO, and GLU, respectively, as described previously. CP and the freeze dried Osborne fractions were determined by the Kjeldahl method according to GB 5009.5-2025 using a nitrogen-to-protein conversion factor of 6.25 and were used as estimates of protein purity. The lyophilized fraction yield was calculated as the mass of the recovered freeze dried fraction relative to the initial mass of defatted pomegranate seed flour. Protein recovery was calculated based on the amount of protein recovered in each fraction relative to the initial protein content of the defatted flour. The extraction yield, protein purity, and protein recovery of CP, ALB, GLO, PRO, and GLU were summarized in Table S3. All freeze-dried samples were stored at −20 °C until further analysis.

### 2.3 Average particle size and zeta-potential

The particle size and zeta-potential were determined using a Litesizer 500 at 25 °C according to Zhang, Boateng, Xu, and Zhang (2024), with slight modifications. The particle size was measured by dynamic light scattering, and the zeta potential was determined using folded capillary cells.

### 2.4 Surface hydrophobicity

Surface hydrophobicity was determined using the ANS fluorescence probe method according to Ma, Mondor, et al. (2025), with slight modifications. Each protein sample (CP, ALB, GLO, PRO, and GLU) was diluted to six concentrations (0.01, 0.02, 0.04, 0.06, 0.08, and 0.10 mg/mL). Fluorescence intensity was recorded at an excitation wavelength of 390 nm and an emission wavelength range of 350–500 nm, and surface hydrophobicity was calculated from the slope of the fluorescence intensity against the protein concentration.

### 2.5 Thermal stability

Thermal stability was evaluated by differential scanning calorimetry (DSC). Samples were heated from 20 to 200 °C under a nitrogen atmosphere, and the onset temperature (T₀), denaturation temperature (Td), and denaturation enthalpy (ΔH) were obtained from the DSC thermograms.

### 2.6 Sodium dodecyl sulfate polyacrylamide gel electrophoresis (SDS-PAGE) analysis

The molecular profiles of PSP and its fractions were analyzed by SDS-PAGE under reducing and non-reducing conditions. Protein samples were mixed with SDS loading buffer with or without DTT, heated, and loaded onto polyacrylamide gel. Protein bands were visualized by Coomassie Brilliant Blue G250 staining.

### 2.7 FTIR spectroscopy

Secondary structural changes were analyzed by attenuated total reflectance Fourier-transform infrared spectroscopy (ATR-FTIR). Spectra were collected in the range of 4000–400 cm^−1^, and the amide I band was deconvoluted for secondary structure estimation.

### 2.8 Circular dichroism spectroscopy

Far-UV circular dichroism (CD) spectra were recorded to further evaluate the secondary structure of PSP and its fractions. Protein samples were dissolved in phosphate buffer, filtered, and scanned from 180 to 260 nm under nitrogen protection conditions.

### 2.9 Protein quality assessment

The amino acid composition was determined by high-performance liquid chromatography (HPLC) after acid hydrolysis. The amino acid contents were expressed as nmol/g protein. Essential amino acid content (EAA), amino acid score (AAS), chemical score (CS), and essential amino acid index (EAAI) were calculated according to Manzanilla-Valdez et al. (2024), with slight modifications, using the following equations:

(1) AAS=AAtAAr

(2) EAA=His+Lys+Met+Phe+Thr+Trp+Val

(3) $$\mathrm{CS}=\frac{AA_{\lim}}{AA_{r}}\times 100$$

(4) $$\text{EAAI}\;\left(\%\right)=\left(\frac{\mathrm{EAA}}{\mathrm{TAA}}\right)\times 100$$

where EAA represents the total essential amino acid content, and TAA represents the total amino acid content. ${\mathrm{AA}}_{t}$ is the content of each essential amino acid in the test protein, ${\mathrm{AA}}_{r}$ is the corresponding amino acid content in the reference amino acid pattern, and $AA_{\lim}$ is the content of the first limiting amino acid in the test protein. The limiting amino acid was identified as the essential amino acid with the lowest AAS or CS value.

### 2.10 In vitro digestion and digestibility determination

In vitro digestion was performed using a simulated gastrointestinal digestion model, as described by Brodkorb et al. (2019), with slight modifications. The protein dispersions were sequentially subjected to salivary, gastric, and intestinal digestion at 37 °C. After each digestion stage, the particle size and zeta potential were determined as described in Section 2.4. In vitro protein digestibility was evaluated by measuring the soluble protein concentration in the digesta using the BCA method and calculated relative to the initial protein concentration.

(5) $$\begin{array}{c} \text{In vitro digestibility}\left(\%\right)=\frac{C_{t}}{C_{0}}\times 100\% \end{array}$$

where C_t_ is the soluble protein concentration in the digesta after each digestion stage and C_0_ is the initial protein concentration before digestion. All measurements were performed in triplicates.

### 2.11 Determination of antinutritional factors

Trypsin inhibition rate, tannin content, and phytic acid content were determined to evaluate the antinutritional characteristics of different pomegranate seed protein fractions (Manzanilla-Valdez et al., 2024). Trypsin inhibition rate was determined based on the inhibition of BAPNA hydrolysis, with absorbance measured at 405 nm. Tannin content was determined after extraction with 75% dimethylformamide and color development with ferric ammonium citrate at 525 nm. Phytic acid content was determined using the ferric ion–sulfosalicylic acid colorimetric method at 500 nm.

### 2.12 Functional properties

The functional properties of PSP fractions, including protein solubility, water-holding capacity (WHC), oil-holding capacity (OHC), emulsifying activity index (EAI), emulsifying stability index (ESI), foaming capacity (FC), and foaming stability (FS), were determined according to Jiménez-Munoz et al. (2023), with slight modifications. The calculation formula is as follows:

(6) $$\text{Solubility}\left(\%\right)=\frac{C_{s}}{C_{t}}\times 100$$

where C_s_ is the protein concentration in the supernatant, and C_t_ is the total protein concentration in the initial dispersion.

(7) $$\begin{array}{c} \mathrm{WHC}=\frac{W_{2}-W_{I}}{W_{0}} \end{array}$$

(8) $$\begin{array}{c} \mathrm{OHC}=\frac{W_{2}-W_{1}}{W_{0}} \end{array}$$

where W_0_ is the weight of the protein sample, W_1_ is the weight of the centrifuge tube plus the dry sample, and W_2_ is the weight of the centrifuge tube plus the hydrated or oil-bound sample after centrifugation.

(9) $$\begin{array}{c} \mathrm{EAI}\left(m^{2}/g\right)=\frac{2\times 2.303\times A_{0}\times \mathrm{DF}}{C\times \varphi \times 10^{4}} \end{array}$$

A₀ is the absorbance at 0 min, DF is the dilution factor, C is the initial protein concentration (0.01 g/mL), and ϕ is the oil volume fraction (0.25).

(10) $$\begin{array}{c} \mathrm{ESI}\left(\min\right)=\frac{A_{0}\times \mathrm{\Delta t}}{A_{0}-A_{10}} \end{array}$$

Δt = 10 min; A₁₀ is the absorbance at 10 min.

(11) $$\mathrm{FC}\left(\%\right)=\frac{V_{1}-V_{0}}{V_{0}}\times 100$$

(12) $$\mathrm{FS}\left(\%\right)=\frac{V_{30}}{V_{1}-V_{0}}\times 100$$

where V_0_ is the initial volume of the protein dispersion before whipping, V_1_ is the volume immediately after whipping, and V_t_ is the volume after standing for t min.

### 2.13 Proteomics analysis

Proteomic profiling was performed using DIA-MS. Proteins were lysed, digested by the FASP method, desalted, and subjected to LC–MS/MS analysis. Raw data were searched against the *Punica granatum* proteome database with a false discovery rate of <1% at both the peptide and protein levels. Differentially abundant proteins were identified using *|*log*₂FC|* ≥ 1 and *P* < 0.05. PCA, Venn diagrams, volcano plots, GO annotation, and KEGG pathway analysis were used to characterize the proteomic differences among the PSP fractions.

### 2.14 Statistical analysis

All experiments were performed in triplicate, and the results are presented as mean ± standard deviation (SD). Statistical analysis was conducted using SPSS 27.0, including one-way ANOVA followed by Tukey's post-hoc test (*P* < 0.05) and Pearson correlation analysis.

## 3 Results and discussion

### 3.1 Average particle size and zeta-potential

The particle size was measured to evaluate the colloidal stability of the protein particles (Zhang, Zhang, Liang, et al., 2024). As shown in Table 1, the particle sizes of PSP fractions differed significantly, ranging from 776.73 ± 25.04 nm to 1585.79 ± 149.02 nm. Among the fractions, ALB exhibited the largest particle size, whereas PRO exhibited the smallest particle size. The larger size of ALB was attributed to the hydration behavior of water-soluble proteins and their tendency to form loose aggregates in aqueous systems. In contrast, the compact size of PRO was associated with its high hydrophobic amino acid content, which favored intramolecular hydrophobic association and limited excessive hydration-driven swelling. A similar trend was reported by Zhao et al. (2025), who observed that prolamin and glutelin fractions from millet bran had smaller particle sizes than albumin and globulin, with prolamin exhibiting a relatively narrow particle size distribution.

**Table 1:** Particle size, zeta potential, surface hydrophobicity, hydrophobic amino acid content, and thermal properties of different PSP fractions.

Protein sample	Particle size (nm)	Zeta potential (mV)	Surface hydrophobicity	Hydrophobic amino acid content (%)	T0 (°C)	△H (J/g)	Td (°C)
ALB	1585.79 ± 149.02^a^	−3.68 ± 0.19^c^	357.27 ± 4.15^b^	11.03 ± 0.24^c^	145.2 ± 1.22^a^	167.19 ± 3.41^b^	165.0 ± 0.31^a^
GLO	1422.96 ± 58.75^ab^	−4.99 ± 0.26^c^	73.23 ± 12.29^d^	9.58 ± 0.81^d^	104.0 ± 0.86^c^	167.98 ± 4.24^b^	150.8 ± 0.78^b^
PRO	776.73 ± 25.04^d^	−5.75 ± 0.50^c^	472.62 ± 6.16^a^	76.35 ± 0.75^a^	87.1 ± 2.20^d^	13.936 ± 1.33^d^	115.1 ± 1.77^c^
GLU	1340.05 ± 139.81^bc^	−30.14 ± 0.2^a^	218.63 ± 8.76^c^	52.04 ± 0.88^b^	99.4 ± 3.64^c^	201.65 ± 4.85^a^	163.2 ± 2.59^a^
CP	1141.64 ± 156.44^cd^	−13.78 ± 0.72^b^	207.23 ± 6.19^c^	11.02 ± 0.27^c^	133.0 ± 0.58^b^	67.277 ± 0.92^c^	149.8 ± 0.53^b^

Note: T0, onset temperature; ΔH, denaturation enthalpy; Td, denaturation temperature. Values with different letters (a-d) in a column are significantly different at *P*<0.05.

Zeta potential was determined to assess the surface charge of protein particles and its relation to electrostatic repulsion and dispersion stability (Zhang, Zhang, Millán Cotto, et al., 2024). As shown in Table 1, the zeta potential values differed markedly among PSP fractions. GLU exhibited the highest absolute zeta potential (−30.14 ± 0.20 mV), whereas ALB, GLO, and PRO displayed much lower values, ranging from −3.68 ± 0.19 mV to −5.75 ± 0.50 mV. The stronger surface charge of GLU was associated with alkaline extraction, which promoted protein unfolding and exposed ionizable groups on the molecular surface. In contrast, the lower zeta potential values of ALB, GLO, and PRO suggested weaker electrostatic repulsion and greater susceptibility to aggregation in aqueous dispersions. Soto-Madrid et al. (2023) reported similar fraction dependent charge differences for chickpea protein fractions, where different protein fractions exhibited distinct zeta potential profiles and dispersion stability. Osborne fractionation revealed marked differences in colloidal characteristics among PSP fractions, providing a basis for understanding their subsequent functional performance in food systems.

### 3.2 Surface hydrophobicity

As shown in Table 1, both surface hydrophobicity and hydrophobic amino acid content differed significantly among PSP fractions (*P* < 0.05). Surface hydrophobicity ranged from 73.23 ± 12.29 to 472.62 ± 6.16, and hydrophobic amino acid content ranged from 9.58 ± 0.81% to 76.35 ± 0.75%. Among the fractions, PRO exhibited the highest surface hydrophobicity and hydrophobic amino acid content. In contrast, GLO exhibited the lowest values for both indices.

The high surface hydrophobicity of PRO was mainly attributed to its alcohol-soluble nature. During ethanol extraction, partial protein unfolding might have exposed nonpolar side chains. This exposure increased the accessibility of hydrophobic patches on the molecular surface. The high hydrophobic amino acid content of PRO further supported this structural feature. Zhang, Boateng, Xu, and Zhang (2024) reviewed plant protein structures and reported that prolamins were alcohol-soluble proteins enriched in hydrophobic residues. This was consistent with the high surface hydrophobicity of PRO observed after ethanol extraction.

The low surface hydrophobicity of GLO indicated limited exposure of nonpolar groups. Its hydrophobic domains might have remained buried within the molecular interior. This structure was consistent with its lower hydrophobic amino acid content and salt-soluble characteristics. GLO might have maintained a more compact conformation under the experimental conditions. These results indicated that surface hydrophobicity was strongly dependent on PSP fraction type. PRO showed greater exposure of nonpolar regions, whereas GLO maintained limited hydrophobic group exposure. This difference reflected the distinct solubility characteristics and conformational states generated during Osborne fractionation.

### 3.3 Protein thermal stability

As shown in Table 1, ALB and GLU exhibited the highest thermal stability among the protein fractions, as indicated by their relatively high T₀, Td, and ΔH values. ALB had the highest T₀ value (145.2 °C), suggesting greater resistance to initial thermal unfolding. In comparison, GLU showed the highest Td (163.2 °C) and ΔH (201.65 J/g), indicating that more thermal energy was required for denaturation and suggesting a more stabilized protein structure. Similar thermal behaviors have been reported for seed storage proteins containing globulin-like components. Similar thermal behaviors have been reported for seed storage proteins containing globulin-like components. This behavior may be related to the ordered quaternary structures of 11S globulin-like proteins, in which acidic and basic subunits are linked by disulfide bonds, thereby enhancing thermal resistance (Ma, Li, et al., 2025; Tang et al., 2024). The high Td and ΔH values of GLU observed in this study are consistent with these results.

These observations suggested that thermal stability might be governed by underlying structural features at the molecular level. Desantis et al. (2022) analyzed protein datasets with experimental melting temperature data and showed that thermal stability was closely associated with residue organization and intramolecular interactions. The high Td and ΔH values of GLU might reflect stronger molecular interactions within this fraction. CP exhibited a relatively high T₀ of 133.0 °C but a markedly lower ΔH, possibly due to its multicomponent nature and overlapping thermal transitions. This interpretation was consistent with the broad SDS-PAGE band distribution, intermediate particle size, and moderate zeta potential, indicating that CP retained heterogeneous protein populations rather than a single dominant thermally stable structure.

### 3.4 SDS-PAGE

As shown in Fig. S1A, the fractionated PSP fractions displayed distinct SDS-PAGE patterns under reducing conditions, reflecting differences in molecular weight distribution. ALB showed several intense bands between 10 and 20 kDa, indicating the predominance of small, water-soluble proteins. GLO exhibited the most complex pattern, with a strong band at around 20 kDa and additional bands between 35 and 70 kDa, which suggested diverse globulin subunits. PRO showed several bands distributed in both the low- and intermediate-molecular-weight regions. GLU displayed relatively weak and diffuse bands, indicating heterogeneous protein components under alkaline extraction conditions (Guzmán-Lorite et al., 2022). CP exhibited multiple bands across a broader molecular weight range, reflecting the coexistence of various protein components in the unfractionated protein sample.

Under non-reducing conditions (Fig. S1B), the electrophoretic profiles changed markedly compared with those under reducing conditions. High-molecular-weight protein components were particularly evident in ALB, PRO, and CP. ALB showed additional components in the high-molecular-weight region, while PRO exhibited distinct bands at approximately 70 kDa and above 130 kDa. CP also showed pronounced protein accumulation in the upper region of the gel. In contrast, these high-molecular-weight components were markedly weakened or absent under reducing conditions, accompanied by the appearance or increased intensity of lower-molecular-weight bands. These changes suggested that disulfide bonds contributed to the association of protein subunits and the formation of higher-molecular-weight protein structures, consistent with recent reducing/non-reducing SDS-PAGE observations of Osborne-fractionated seed proteins in which high-molecular-weight assemblies were associated with interchain disulfide bonds (Bojórquez-Velázquez et al., 2026). These fraction-specific electrophoretic features were supported by prior SDS-PAGE analyses of PSP, where major bands at 18 kDa and below 10 kDa were observed and identified as 2S albumins and other proteins (Tuppo et al., 2024). Similar profiles were reported for PSP extracts and hydrolysates (Rahimipanah et al., 2022).

### 3.5 Secondary structural characteristics of different pomegranate seed protein fractions

As shown in Fig. 1A, CP exhibited a broad amide A band at 3295 cm^−1^, which was mainly associated with O-H/N-H stretching vibrations from protein and polysaccharide-related groups (Guo et al., 2025; Hong et al., 2021). ALB showed pronounced absorption peaks at 3366 and 1073 cm^−1^, which suggested the presence of abundant hydroxyl-containing groups, possible glycosylated structures, or protein–polysaccharide associations. These spectral features were consistent with its strong hydrophilicity and hydration capacity (Hong et al., 2021). In contrast, GLU displayed a characteristic absorption peak at 2962 cm^−1^, which corresponded to the asymmetric stretching of methyl-containing groups and indicated greater exposure of hydrophobic amino acid side chains. This result was consistent with its relatively high surface hydrophobicity. Compared with CP, fractionated PSP samples showed clearer amide-related bands and weaker non-protein signals, indicating improved separation of solubility-defined protein fractions and reduced interference from residual polysaccharides and lipids (Zhao et al., 2025). Weak carbonyl absorption peaks were still observed in CP, GLO, and PRO, suggesting the presence of trace lipid-associated residues. Their absence in ALB and GLU indicated fraction-dependent differences in minor non-protein components.

**Fig. 1:**
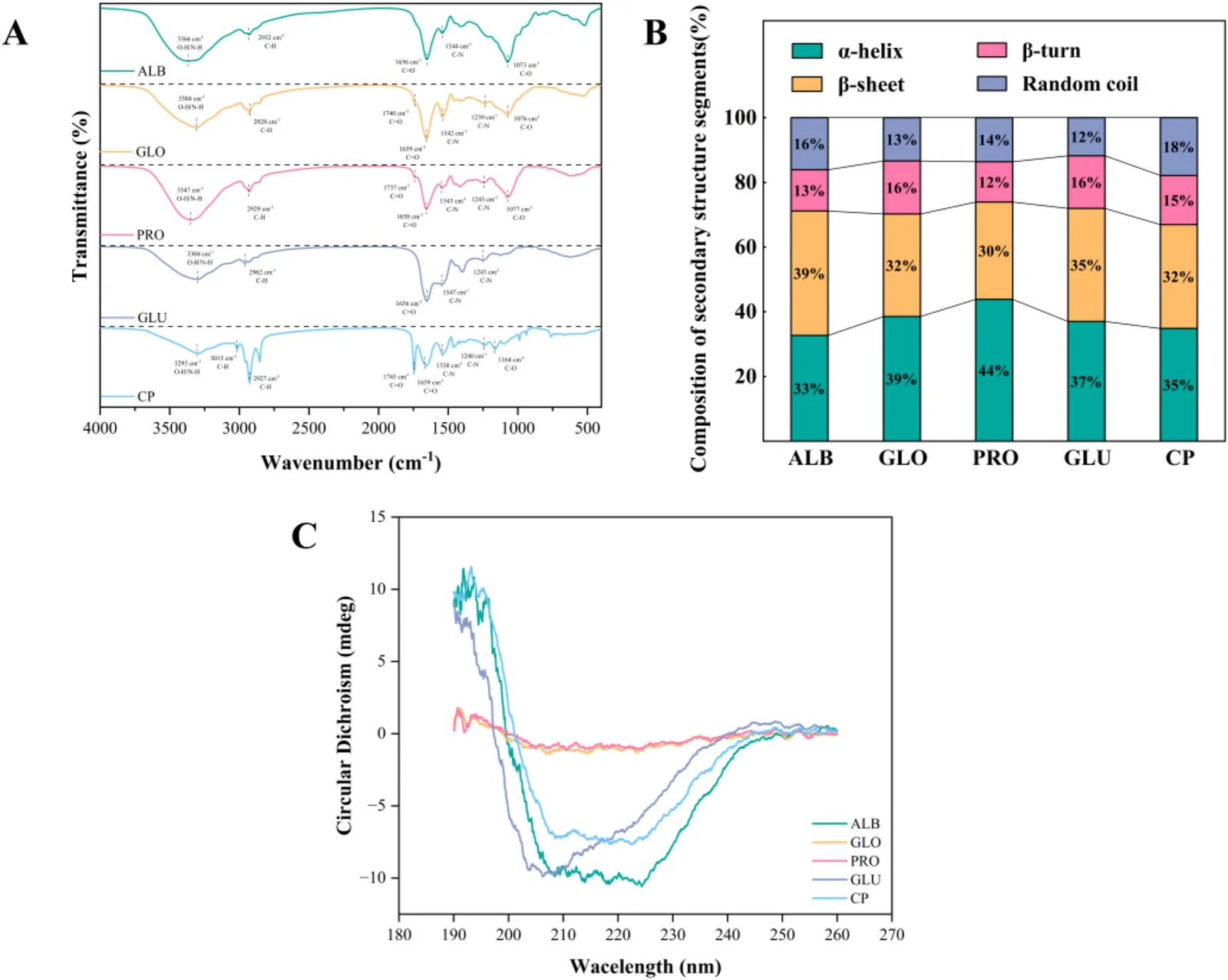
Structural characterization of different PSP fractions and their crude extracts: FTIR spectra (4000–400 cm ^− 1^) (A), protein secondary structure content distribution (B), and circular dichroism spectra (180–270 nm) (C).

The amide I region at 1700–1600 cm^−1^ was further analyzed to evaluate secondary structural differences among PSP fractions. As shown in Fig. S2 and Fig. 1B, the quantitative results showed clear fraction-dependent differences in secondary structure composition. GLO contained 39% α-helix and 32% β-sheet, while GLU contained 37% α-helix and 35% β-sheet. These relatively high ordered-structure contents might be related to their salt- and alkali-soluble protein composition. They also suggested stronger intra- and intermolecular interactions in GLO and GLU. Previous FTIR studies have shown that changes in α-helix and β-sheet contents could reflect differences in protein folding and molecular association (Soto-Madrid et al., 2023). ALB showed the highest β-sheet content, indicating stronger interchain hydrogen bonding and greater backbone rigidity (Shivu et al., 2013). This structural feature was consistent with its high thermal transition temperature, particularly the highest T₀ value observed in Section 3.4. β-sheet enrichment had also been associated with stronger hydrogen bonding in intermolecular β-sheet structures. CP showed the highest random coil content (18%), reflecting the conformational heterogeneity of the crude multicomponent protein extract. This result also agreed with the markedly lower ΔH of CP, suggesting broader structural diversity but lower structural uniformity than the isolated PSP fractions. Composition-dependent structural variation had also been reported in established plant proteins. For example, soybean protein isolates from different cultivars contained 19.45–26.28% α-helix and 33.97–39.61% β-sheet, with variations in 7S/11S composition associated with differences in protein structure and thermal stability (Ma, Mondor, et al., 2025). Compared with these reported structural profiles, the PSP fractions exhibited a distinct secondary-structure distribution, particularly the relatively high α-helix proportions observed in GLO, GLU, and PRO.

PRO showed the highest α-helix content among all PSP fractions, reaching 44%, compared with 33% in ALB, 39% in GLO, 37% in GLU, and 35% in CP. Its β-sheet content was 30%, which was lower than those of ALB, GLO, GLU, and CP. This result indicated that PRO was characterized by a higher proportion of α-helical conformation. Shah et al. (2024) reported that prolamin-type proteins commonly contain hydrophobic domains and locally ordered α-helical segments. This supported the association between the high α-helix content of PRO and its strong hydrophobic characteristics.In the present study, the high α-helical proportion, together with the strong surface hydrophobicity and low thermal stability of PRO, suggested that its structure was locally ordered but less thermally stable.

As shown in Fig. 1C, all samples exhibited positive signals near 190–195 nm and negative bands at approximately 208 and 222 nm, indicating the presence of α-helical conformations in the protein structure. The more distinct double-negative bands of ALB, GLO, and GLU suggested that these fractions maintained relatively ordered conformations in solution, whereas the flatter spectrum of CP reflected greater conformational flexibility and disorder. Although PRO showed the highest α-helix content based on FTIR analysis, its negative CD bands at approximately 208 and 222 nm were relatively weak. This discrepancy might have been related to its local conformational characteristics. Considering its amino acid composition, the relatively high Ala content might have favored local α-helical structures, whereas the cyclic pyrrolidine structure of Pro restricted the conformational freedom of the polypeptide backbone, potentially which potentially contributed to greater local conformational heterogeneity (Umumararungu et al., 2024). Overall, the CD results were consistent with the FTIR-derived secondary structure analysis, confirming that ALB, GLO, and GLU retained more ordered conformations, whereas CP exhibited greater structural disorder. These results suggested that the distinct chemical environments, secondary structural organization, and aggregation states of the different pomegranate seed protein fractions were closely associated with their intrinsic protein composition and solubility characteristics. These fraction-specific structural features might further explain the differences in thermal stability, surface hydrophobicity, interfacial functionality, and digestion behavior among fractions. The discrepancy between the fitted α-helix content and raw CD spectral intensity of PRO further indicated that its highly hydrophobic nature and heterogeneous dispersion state might contribute to its unique structural and functional behavior.

### 3.6 Functional properties

The amino acid composition and spatial conformation of the polypeptide chains directly determined the hydrophilic–hydrophobic balance on the protein surfaces, which subsequently dictated their affinities in aqueous or lipid systems. As shown in Fig. 2A and B, ALB exhibited the highest solubility, reaching 24.32 ± 0.80%, and the lowest OHC, at 0.41 ± 0.06 g/g. PRO showed the lowest solubility (1.35 ± 0.25%) and the highest OHC (2.32 ± 0.20 g/g), followed by GLO (2.21 ± 0.16 g/g). The high solubility of ALB suggested stronger water affinity, whereas the high OHC of PRO and GLO indicated greater lipid-binding capacity under the experimental conditions. These differences might be related to the different hydrophilic and hydrophobic characteristics of the protein fractions. Proteins with stronger water affinity tend to show higher solubility, whereas proteins with stronger hydrophobic characteristics were more likely to bind to oil (Zhang et al., 2023). The surface of ALB was rich in polar amino acid residues, which readily formed dense hydrogen-bond networks with water molecules, thereby achieving high solubility (Ghorbani et al., 2025). In contrast, the conformation of PRO buried or exposed numerous hydrophobic microdomains. Although this structural feature restricted its dispersion in the aqueous phase, it promoted its hydrophobic interactions with lipid molecules. These findings suggested that ALB might have had greater potential for water dispersible systems, whereas PRO might have been of interest for lipid rich formulations.

**Fig. 2:**
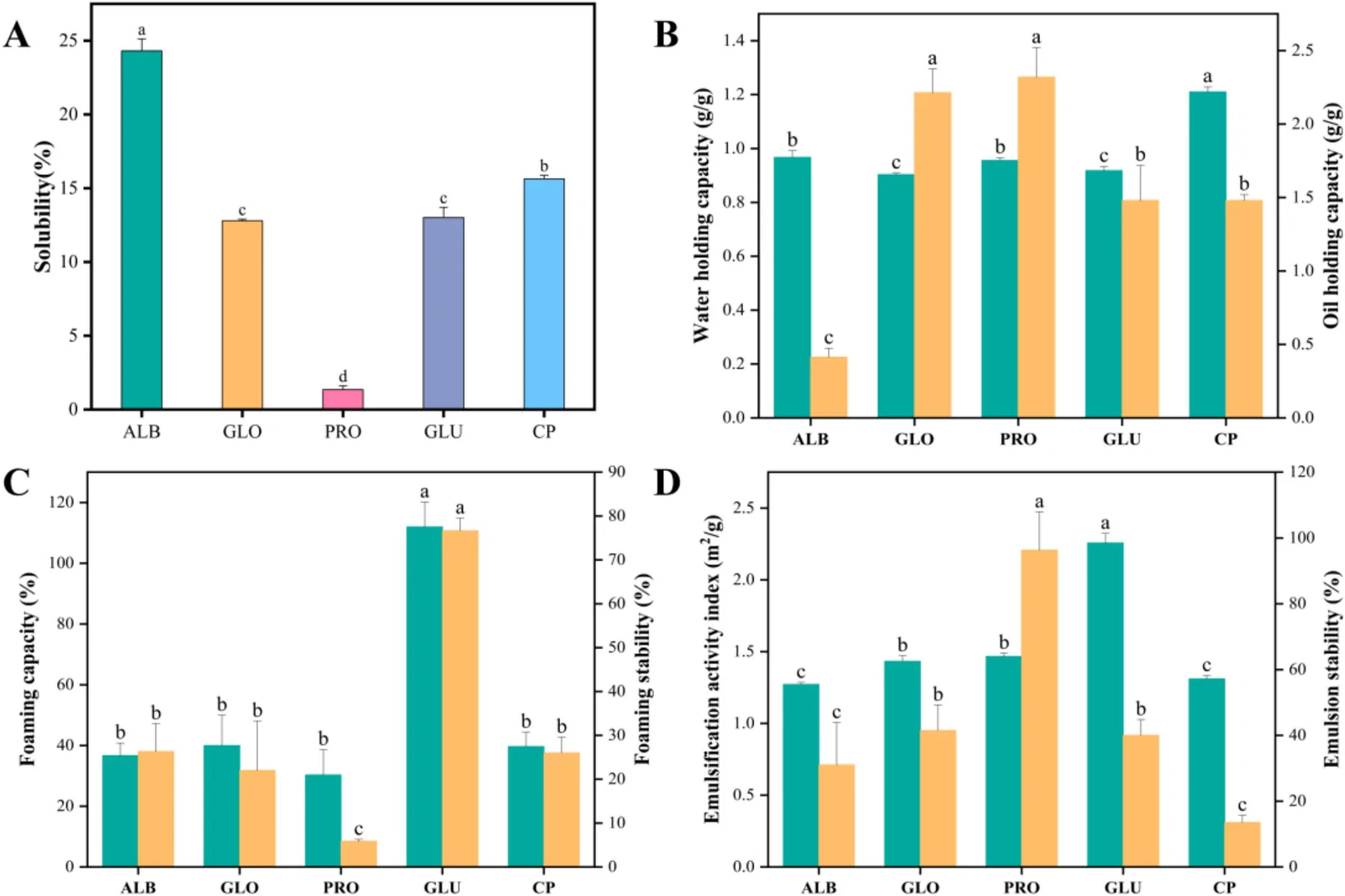
Techno-functional properties of PSP fractions and CP. (A) Protein solubility; (B) WHC and OHC; (C) EAI and ESI; and (D) FC and FS. Data are expressed as mean ± standard deviation (*n* = 3). Different lowercase letters indicate significant differences between samples (*P* < 0.05).

Superior foaming performance did not entirely rely on high solubility; rather, it was closely associated with the ability of proteins to adsorb and rearrange at the air–water interface to form protective interfacial films (Zhang et al., 2025). As shown in Fig. 2C, although the solubility of GLU was only at an intermediate level (13.02 ± 0.68%), its FC and FS reached 112.00 ± 8.19% and 76.67 ± 2.89%, respectively, which were higher than those of ALB (36.67 ± 4.16% and 26.33 ± 6.35%). This behavior was consistent with the findings of Zhao et al. (2020), who reported that structural modifications of rice glutelin, such as partial unfolding and exposure of flexible molecular regions, improved its solubility, interfacial adsorption, foaming capacity, and foam stability. These changes were mainly attributed to enhanced molecular flexibility and increased exposure of surface-active groups, which facilitated protein adsorption and rearrangement at the air–water interface. During foaming, moderately dissociated GLU migrate to the air–water interface and participate in the formation of a compact interfacial film through molecular rearrangement and possible disulfide bond interactions. This interfacial film might provide steric protection around air bubbles and delay bubble coalescence, thereby contributing to the excellent foaming stability of GLU.

As shown in Fig. 2D, GLU had the highest EAI among all PSP fractions, with a value of 2.26 ± 0.07 m^2^/g. PRO showed the lowest solubility, but its ESI reached 96.33 ± 11.58 min, which was the highest among all fractions. The difference between EAI and ESI reflected the distinct emulsification mechanisms of different PSP fractions. EAI was primarily associated with the rapid adsorption of proteins at the oil–water interface and the reduction of interfacial tension, whereas ESI was more closely related to the strength and stability of the interfacial layer formed around oil droplets (Zhang et al., 2023). At the tested pH, the relatively flexible structural domains of GLU might have facilitated its rapid adsorption at the oil–water interface, thereby contributing to its high EAI. In contrast, the strong hydrophobicity of PRO might have promoted stronger protein–protein and protein–oil interactions at the interface, resulting in a more stable interfacial layer and the highest ESI. Similar relationships between protein structural properties and emulsifying behavior have been reported in recent studies on plant protein emulsifiers (Lima et al., 2024). Therefore, the functional profiles of ALB, PRO, and GLU suggested their potential for aqueous dispersion, lipid-rich or emulsion-stabilizing, and aerated systems, respectively. These potential applications should have been further evaluated in model and practical food formulations. The functional performance of several PSP fractions also compared favorably with values reported for established plant protein ingredients. PRO exhibited an OHC of 2.32 ± 0.20 g/g, which was higher than the approximately 0.8–1.7 g/g reported for most commercial soy, pea, faba bean, chickpea, mung bean, oat, and wheat protein ingredients (Jakobson et al., 2023). Moreover, the FC of GLU reached 112.00 ± 8.19%, exceeding the values of 98% and 95% reported for commercial wheat and potato proteins, respectively, in the same study. PRO also showed a high ESI of 96.33 ± 11.58 min, comparable to or higher than values recently reported for soy (91.7 min) and pea (48.9 min) proteins (Pilla et al., 2025). Taken together, these comparisons suggested that specific PSP fractions possessed useful oil-binding, foaming, and emulsion-stabilizing properties that might have supported their application in selected food systems.

### 3.7 Protein quality assessment

The Osborne fractionation approach effectively separated PSP into fractions with distinct amino acid profiles (Table 2). Among the tested fractions, ALB showed the most favorable nutritional profile, with significantly higher concentrations of all EAAs than the other fractions and CP (*P* < 0.05). Accordingly, ALB had the highest EAA content, AAS, and EAAI, with an EAAI value of 15.24%, suggesting its relatively higher nutritional potential among the PSP fractions. Commercial soy, pea, and faba bean proteins were also reported to exhibit source-dependent differences in essential amino acid composition and limiting amino acids (Auer et al., 2024). In this context, the higher EAA-related indices of ALB among the PSP fractions suggested that Osborne fractionation could selectively enrich a fraction with a relatively favorable essential amino acid profile. A similar effect of fractionation on nutritional quality was reported by Ma, Li, et al. (2025), who found that specific Osborne fractions from *Alphitobius diaperinus* contained higher EAA levels and showed improved biological values compared with the crude extract.

**Table 2:** Amino acid composition analysis of different PSP fractions.

Amino Acid(nmol/g protein)		ALB	GLO	PRO	GLU	CP
EAAs	His	3864.1 ± 94.03^a^	272.71 ± 6.22^b^	270.16 ± 6.21^b^	228.23 ± 20.48^b^	24.17 ± 1.64^c^
	Lys	5676.53 ± 175.41^a^	3121.11 ± 103.1^b^	593.95 ± 23.36^c^	58.35 ± 4.07^d^	42.56 ± 2.37^d^
	Met	1584.27 ± 112.15^a^	1061.61 ± 28.32^b^	114.61 ± 5.51^c^	61.93 ± 5.51^cd^	12.69 ± 0.61^d^
	Phe	3032.37 ± 110.94^a^	1380.99 ± 78.9^b^	400.6 ± 16.19^c^	50.4 ± 3.82^d^	21.76 ± 1.01^d^
	Thr	5490.07 ± 106.65^a^	2342.14 ± 474.2^b^	385.24 ± 112.02^c^	154.89 ± 20.4^c^	54.29 ± 3.42^c^
	Trp	3656.79 ± 127.2^a^	288.03 ± 30.33^c^	898.5 ± 29.88^b^	160.14 ± 19.91^d^	38.85 ± 1.22^e^
	Val	6892.81 ± 476.37^a^	1638.04 ± 43.92^b^	668.02 ± 26.31^c^	94.45 ± 10.26^d^	29.71 ± 1.22^d^

NEAAs	Ala	4601.13 ± 23.66^b^	69,176.93 ± 12358^a^	71,943.77 ± 13,802.06^a^	90.9 ± 14.48^b^	190.42 ± 9.34^b^
	Arg	0	2387.07 ± 70.38^a^	1893.93 ± 100.32^b^	503.73 ± 65.82^c^	182.2 ± 5.82^d^
	Asp	40,435.6 ± 752.38^a^	4551.69 ± 283.37^c^	9810.12 ± 841.06^b^	544.57 ± 43.41^d^	373.88 ± 20.7^e^
	Glu	19,642.13 ± 1679^a^	3616.85 ± 837.77^b^	1411.5 ± 237.98^d^	2435 ± 176.75^c^	245.16 ± 13.49^e^
	Gly	2668.35 ± 302.27^a^	0	0	544.07 ± 34.56^b^	57.97 ± 4.6^c^
	Pro	9.37 ± 0.33^d^	47,906.9 ± 8251.36^a^	13,600.83 ± 2572.18^b^	44.19 ± 1.66^c^	39.14 ± 2.03^c^
	Ser	25,289.37 ± 1531.67^a^	1873.01 ± 25.09^c^	3202.09 ± 105.52^b^	1047.98 ± 133.02^d^	242.19 ± 12.39^e^
	Tyr	270.14 ± 13.34^b^	580.76 ± 12.57^a^	258.3 ± 10.55^b^	318.55 ± 44.19^b^	21.74 ± 1.27^c^

AAS		2.30(Val)	0.82(Lys)	0.52(Trp)	0.10(Phe)	0.02(Trp)
EAA(mg/g)		4.37	1.41	0.51	0.12	0.03
EAAI (%)		15.24%	8.70%	3.23%	10.32%	8.70%
CS		0.003%(Met)	0.41%(Met)	0.28%(Met)	0.03%(Met)	0.01%(Lys)

Note: Values are expressed as mean ± standard deviation (*n* = 3). Values with different letters (a-e) in a column are significantly different at *P* < 0.05*.*

The separated fractions also exhibited distinct enrichment patterns of characteristic amino acids. For example, GLO and PRO were characterized by markedly high Ala content, reaching approximately 70,000 nmol/g protein, nearly 15-fold higher than that of ALB. PRO showed a highly unbalanced amino acid composition, with abundant Ala and Pro but relatively low EAA content. This compositional feature was consistent with its high hydrophobic amino acid content, compact hydrodynamic diameter, and strong surface hydrophobicity, as discussed in 3.1, 3.2. The enrichment of hydrophobic and structure-related residues in PRO might favor compact molecular packing and hydrophobic interactions, thereby contributing to its low water affinity and functional behavior at oil–water interfaces. Similar structure–function relationships have been observed in millet bran protein fractions, where surface hydrophobicity was closely related to the distribution, structure, and content of hydrophobic residues. The exposure of hydrophobic residues was shown to promote protein–oil interactions and emulsifying properties (Zhao et al., 2025).

Despite the marked differences in amino acid profiles among the fractions, CS analysis revealed clear nutritional limitations. Met was identified as the first limiting amino acid in ALB, GLO, PRO, and GLU, whereas Lys was identified as the limiting amino acid in CP. These results suggested that sulfur-containing amino acid deficiency was the major nutritional constraint for most fractionated PSP samples. This widespread deficiency of sulfur-containing amino acids in plant-derived protein components was consistent with the report by Pérez-Vila et al. (2025), who showed that methionine and cysteine were the primary limiting factors in several quinoa leaf protein varieties despite their otherwise favorable EAA profiles. Therefore, although ALB exhibited the best essential amino acid profile among the tested fractions, its application as a nutritional ingredient might still require supplementation with methionine-rich protein sources to better meet human nutritional requirements.

### 3.8 In vitro digestion characteristics

As shown in Fig. 3A and B, the particle size and zeta potential of PSP fractions underwent pronounced stage-dependent changes during simulated gastrointestinal digestion. During the gastric phase, all protein fractions showed a decrease in the absolute value of zeta potential compared with the salivary phase, indicating weakened electrostatic stabilization. Marked particle enlargement was observed for ALB and PRO, whose particle sizes increased to 1662.83 nm and 1123.56 nm, respectively (*P < 0.05*). This behavior was consistent with previous reports showing that acidic conditions can reduce the absolute zeta potential of proteins, thereby weakening electrostatic repulsion and promoting protein aggregation (Lopez-Sanchez et al., 2020).

**Fig. 3:**
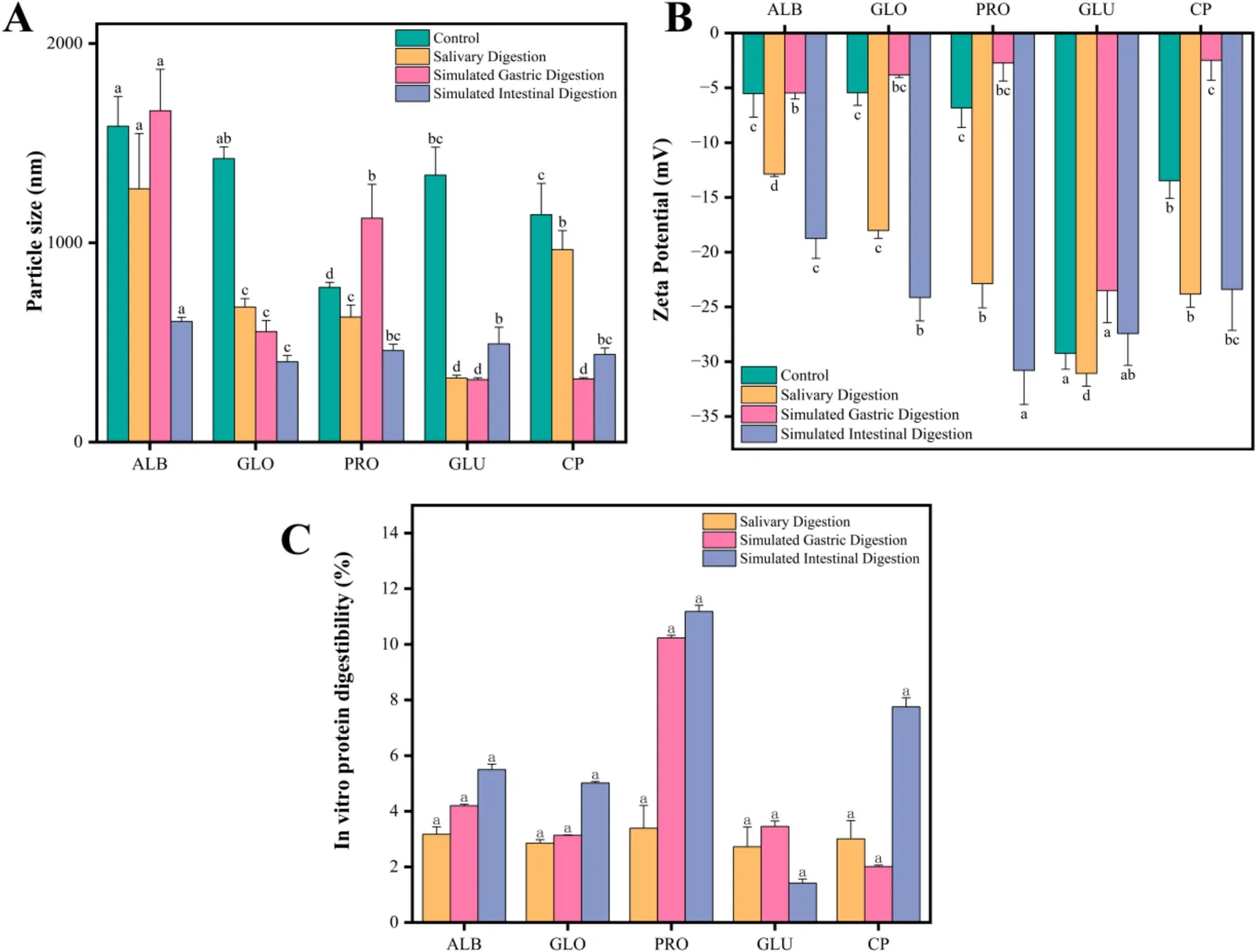
In vitro digestion characteristics of different pomegranate seed protein fractions and their crude extracts. (A) Particle size change; (B) Zeta potential change; and (C) in vitro digestibility. Different lowercase letters indicate significant differences among samples (*P*<0.05).

After transition to the intestinal phase, the zeta potential values shifted toward more negative values, especially for PRO, which reached −30.79 mV. The particle sizes of ALB, GLO, and GLU decreased to 606.03, 403.13, and 493.82 nm, respectively. These changes suggested that intestinal digestion promoted the redistribution of surface charges and partial dissociation of gastric aggregates. The increased negative charge might be associated with the deprotonation of ionizable groups, presence of intestinal salts and bile salts, and generation of smaller peptide fragments by trypsin hydrolysis (Chen et al., 2022). These factors enhance electrostatic repulsion and improve colloidal dispersion during the intestinal stage.

The physicochemical state of each protein fraction was closely related to its enzymatic susceptibility. As shown in Fig. 3C, in vitro digestibility generally increased from the salivary to intestinal stages for most PSP fractions. PRO showed a sharp increase in digestibility from 13.39% in the salivary phase to 20.23% in the gastric phase and reached the highest digestibility after intestinal digestion, with a value of 21.18%. Although PRO exhibited particle enlargement during gastric digestion, this aggregation did not appear to prevent enzymatic hydrolysis. The acidic gastric environment might have promoted partial structural rearrangement and exposed pepsin-accessible cleavage sites, contributing to the relatively high digestibility of PRO. This interpretation was consistent with previous findings that prolamin-type proteins could undergo structural changes under gastric conditions, which might affect their susceptibility to enzymatic digestion (Liu et al., 2019).

GLU showed a decrease in digestibility from 13.45% after gastric digestion to 11.42% after intestinal digestion. The zeta potential became more negative in the intestinal phase (−27.42 mV), while its particle size increased from 313.63 nm in the gastric phase to 493.82 nm in the intestinal phase. This result suggested that GLU might have undergone secondary aggregation or flocculation under intestinal conditions. Such aggregation could reduce the accessibility of trypsin to cleavage sites and thereby limit further hydrolysis. This phenomenon might be related to the relatively compact structure of GLU and the presence of strong intermolecular interactions, such as hydrophobic interactions and possible disulfide-associated aggregation, which could restrict enzyme accessibility (Vargas-Alvarez et al., 2021). The intestinal digestibility of GLU was likely governed not only by surface charge but also by the balance between aggregate dissociation, secondary aggregation, and enzyme-accessible structural domains.

### 3.9 Antinutritional factors

As shown in Table 3, the different pomegranate seed protein fractions exhibited distinct antinutritional profiles. ALB had the highest EAAI (15.24%) and the lowest trypsin inhibition rate (5.08%), indicating a favorable combination of essential amino acid quality and low protease-inhibitory activity. GLO showed an intermediate EAAI (8.70%) but the highest trypsin inhibition rate (16.32%) and phytic acid content (11.28 μmol/g). Trypsin inhibitors could reduce digestive protease activity, while phytic acid might limit mineral bioaccessibility through mineral chelation; therefore, the relatively high levels of these factors should have been considered when evaluating the nutritional quality of GLO (Auer et al., 2024; Wang et al., 2025).

**Table 3:** Trypsin inhibition rate, tannin content, and phytic acid content of different PSP fractions.

Protein fraction	Trypsin inhibition rate (%)	Tannin content (mg/kg)	Phytic acid content (μmol/g wet basis)
CP	11.68 ± 6.45	0.15 ± 0.41	6.03 ± 0.04
ALB	5.08 ± 6.50	57.54 ± 1.20	7.76 ± 0.38
GLO	16.32 ± 6.06	0.04 ± 0.23	11.28 ± 0.34
PRO	9.87 ± 3.80	51.39 ± 2.06	1.03 ± 0.47
GLU	8.31 ± 2.34	64.49 ± 2.09	1.14 ± 0.11

Note: Values are expressed as mean ± standard deviation (*n* = 3).

PRO exhibited the highest intestinal IVPD (21.18%) together with the lowest phytic acid content (1.03 μmol/g), representing a comparatively favorable combination of protein digestibility and low phytate content. In contrast, GLU showed a relatively low intestinal IVPD (11.42%) and the highest tannin content (64.49 mg/kg), although its phytic acid content remained low. Tannins could interact with proteins and digestive enzymes and might have influenced protein utilization, but their nutritional effects depended on concentration and the food matrix (Manzanilla-Valdez et al., 2024; Wang et al., 2025). Overall, ALB exhibited the most favorable overall nutritional profile and might have represented the most promising fraction for future nutritional applications. PRO also showed potential because of its high intestinal digestibility and low phytic acid content, although its unbalanced amino acid composition remained a nutritional limitation.

### 3.10 Proteomic analysis

DIA-based proteomics identified approximately 3900 proteins in the five pomegranate seed protein fractions. PCA revealed clear differences in the global proteomic profiles among the fractions (Fig. 4A), with PC1 and PC2 explaining 39.7% and 16.8% of the total variance, respectively. GLU, GLO, and PRO were positioned relatively close to each other, suggesting partial compositional similarity, whereas ALB and CP showed distinct proteomic signatures. These results indicated that Osborne fractionation substantially reshaped the protein composition of PSP and selectively enriched solubility-defined protein subgroups, a phenomenon commonly observed in plant seed proteomes (Chen et al., 2025).

**Fig. 4:**
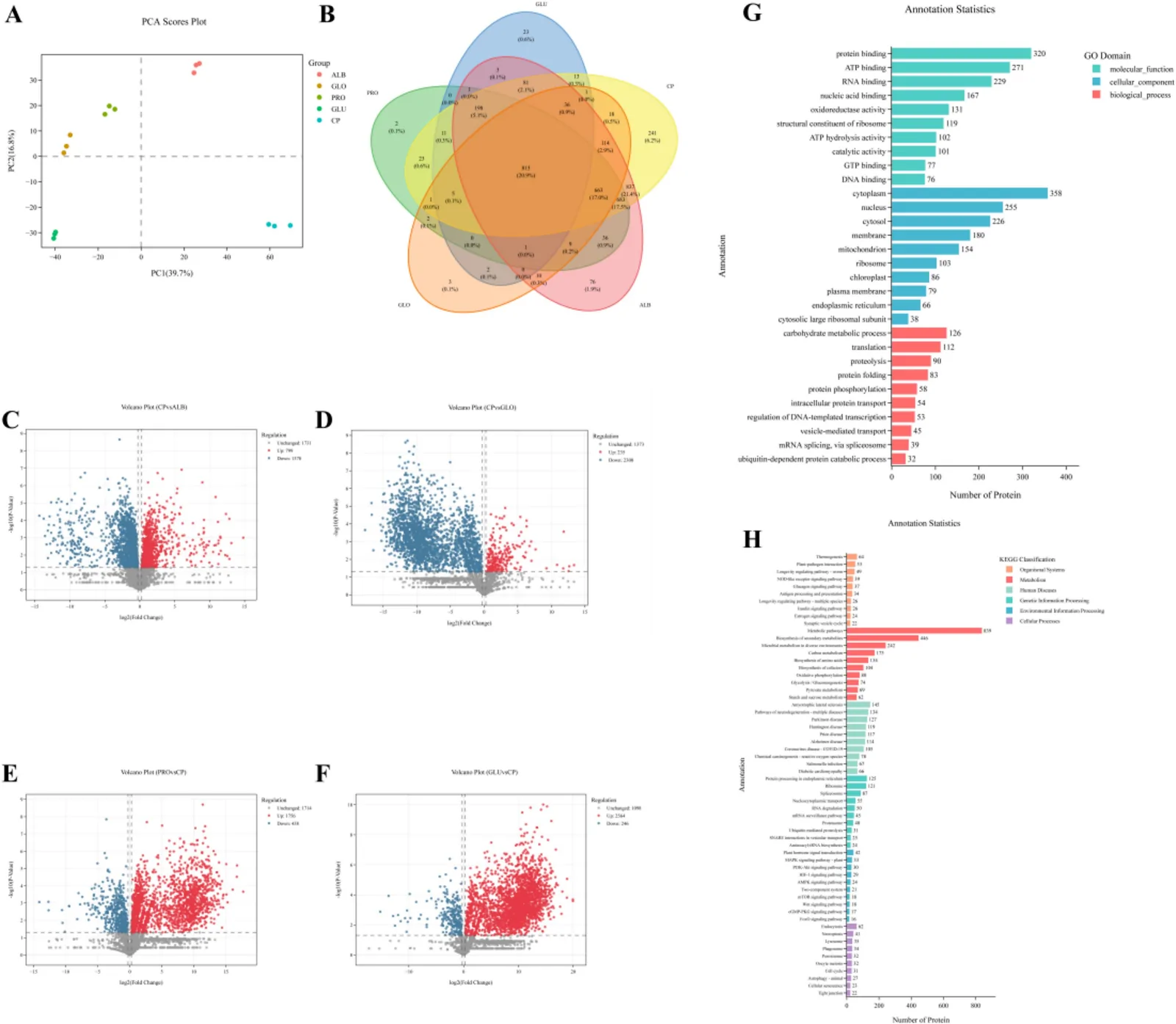
Multidimensional proteomic characterization and differential abundance analysis of PSP and CP fractions. (A) PCA score plot of protein expression profiles; (B) Venn diagram showed the overlap and specificity of the identified proteins; (C–F) Volcano plots illustrated differentially abundant proteins in pairwise comparisons for CP vs. ALB (C), CP vs. GLO (D), PRO vs. CP (E), and GLU vs. CP (F); (G) Gene Ontology (GO) annotation statistics of differentially abundant proteins; and (H) Kyoto Encyclopedia of Genes and Genomes (KEGG) functional pathway classification.

The Venn diagram further supported this fraction-specific distribution (Fig. 4B). Among the identified proteins, 815 were shared across all five fractions, accounting for 20.9% of the total proteins, while each fraction also contained specific proteins. This confirmed that solubility-based fractionation separated and enriched different protein subgroups. Differential abundance analysis using *∣log2FC∣* ≥ 1 and *P* < 0.05 further revealed comparison-specific enrichment patterns among the fractions (Fig. 4C–F). Overall, PRO and GLU showed a larger number of proteins with higher relative abundance than CP, whereas ALB and GLO exhibited distinct enrichment or depletion patterns. These results suggested that Osborne fractionation reconstructed the proteomic composition of each fraction rather than merely concentrating the total proteins, consistent with extraction-driven proteome remodeling reported in other plant protein systems (Yang et al., 2024).

Representative differentially abundant proteins included 2S albumin-like proteins, non-specific lipid-transfer proteins (nsLTPs), bifunctional inhibitor/lipid-transfer protein domain-containing proteins, 11S globulin-like proteins, vicilin-like storage proteins, and cupin domain-containing proteins. These proteins were functionally associated with seed protein storage, lipid interaction, structural stability, and interfacial behavior. Specifically, 2S albumin-like proteins are small water-soluble storage proteins, and their fraction-specific accumulation may be related to the higher solubility and hydration-associated properties of ALB. In contrast, nsLTPs and bifunctional inhibitor/lipid-transfer protein domain-containing proteins are generally associated with lipid binding and transfer. Plant lipid-binding proteins, such as acyl-CoA-binding proteins, have been reported to participate in lipid metabolism and lipid-associated interactions (Du et al., 2016), while plant protein-based emulsifiers stabilize emulsions mainly through interfacial adsorption, surface hydrophobicity, and interfacial film formation (Zhang et al., 2023). Therefore, the enrichment of lipid-transfer-related proteins may help explain the stronger oil-binding capacity and emulsion-stabilizing behavior observed in hydrophobic fractions such as PRO. Moreover, 11S globulin-like proteins, vicilin-like storage proteins, and cupin domain-containing proteins are typical seed storage protein components with compact globular structures and strong intermolecular interactions. Husband et al. (2024) reported that the relative abundance of vicilin- and legumin-type globulins affected protein structure, functionality, and nutritional quality. Composition-dependent effects were also demonstrated in established plant protein systems. In soybean, differences in 7S and 11S storage globulin composition were associated with variations in thermal stability and functional properties, while the relative abundance of 7S vicilin and 11S legumin influenced the functionality and nutritional quality of pea protein (Husband et al., 2024; Ma, Mondor, et al., 2025). In comparison, the distinct distribution of 2S albumin-like proteins, nsLTPs, 11S globulin-like proteins, vicilin-like proteins, and cupin-domain proteins among the PSP fractions provided a compositional basis for their fraction-specific structural and functional characteristics. Thus, the enrichment of these storage proteins may contribute to the ordered secondary structures, aggregation behavior, thermal stability, and amino acid-derived nutritional differences observed among PSP fractions, particularly the higher thermal stability of GLU. In addition, extraction- and fractionation-induced differences in protein aggregation have been shown to affect the techno-functional properties of plant proteins (Yang et al., 2024), which further supported the association between the SDS-PAGE patterns and the functional differentiation of PSP fractions. Therefore, the fraction-specific accumulation of these proteins was consistent with the observed differences in water solubility, surface hydrophobicity, thermal stability, nutritional quality, and functional behavior among PSP fractions, and provided complementary compositional information for interpreting these differences. These relationships were based on protein annotation and relative abundance patterns and did not constitute direct functional or mechanistic validation. Screening of the proteomic dataset identified six potential allergenic proteins in PSP and its Osborne fractions (Table S4), including three Pun g 1 nsLTP isoforms, Pun g 14, and two pomegranate seed 2S albumins. The Pun g 1 isoforms showed higher relative abundance in GLO and PRO than in GLU and CP, whereas Pun g 14 was most abundant in PRO. The two 2S albumins also showed distinct distribution patterns, with Pun g 2S-A1 showing the highest signal in GLO and Pun g 2S-A2 in PRO; both proteins showed lower signals in GLU. These differences might have been related to the distinct physicochemical properties and protein compositions of the Osborne fractions, which influenced the partitioning and retention of individual proteins during fractionation. The detection of Pun g 1 isoforms, Pun g 14, and the two 2S albumins was consistent with the documented allergenic relevance of these pomegranate proteins (O'Malley et al., 2021; Tuppo et al., 2018; Tuppo et al., 2024). These results suggested that Osborne fractionation redistributed, but did not completely remove, potential allergenic proteins among the PSP fractions. However, relative DIA-MS signals did not directly indicate absolute protein content or clinical allergenicity; these properties required confirmation using targeted immunological assays.

GO annotation showed that the differentially abundant proteins were mainly enriched in protein binding, ATP binding, and RNA binding categories (Fig. 4G), suggesting the presence of proteins involved in molecular recognition, energy-dependent processes, protein homeostasis, and post-transcriptional regulation. These GO categories indicated that the proteomic differences among PSP fractions were not limited to storage proteins but also involved proteins participating in synthesis, folding, assembly, transport, and quality control during seed maturation and storage protein accumulation. This was consistent with the reported roles of seed proteomes in protein synthesis, folding, assembly, and quality-control processes (Al-Obaidi et al., 2024; Yang et al., 2023). These protein homeostasis-related processes may also influence conformational flexibility, aggregation state, and interfacial rearrangement, which are important for foaming and emulsifying behavior (Zhang et al., 2025).

KEGG pathway classification further showed that metabolic pathways were dominant, with 839 proteins accounting for more than 60% of the annotated proteins (Fig. 4H). Pathways related to amino acid biosynthesis, ribosomes, protein processing in the endoplasmic reticulum, proteasomes, and ubiquitin-mediated proteolysis were also highly represented. These pathways might have been associated with the observed differences in essential amino acid profiles, thermal stability, digestibility, emulsification, and foaming properties among PSP fractions. The enrichment of proteasome and ubiquitin-mediated proteolysis pathways also suggests the involvement of protein quality-control mechanisms during seed maturation, desiccation, and storage (Linhares & Ramos, 2023; Lopez-Sanchez et al., 2020).

### 3.11 Correlation analysis

As illustrated in Fig. 5, Pearson correlation analysis revealed several key structure–function relationships among the PSP fractions. The secondary structural parameters were closely associated with thermal stability. Ordered structures, especially α-helix and β-sheet, were positively correlated with Td and ΔH, with correlation coefficients ranging from *r* = 0.78 to 0.83, whereas the random coil content was negatively correlated with ΔH (*r* = −0.89). These results suggested that a higher proportion of ordered conformations might strengthen intramolecular interactions and improve resistance to thermal unfolding. Similar relationships between protein secondary structure and thermal stability have been reported in plant protein systems (Kaczmarek et al., 2025).

**Fig. 5:**
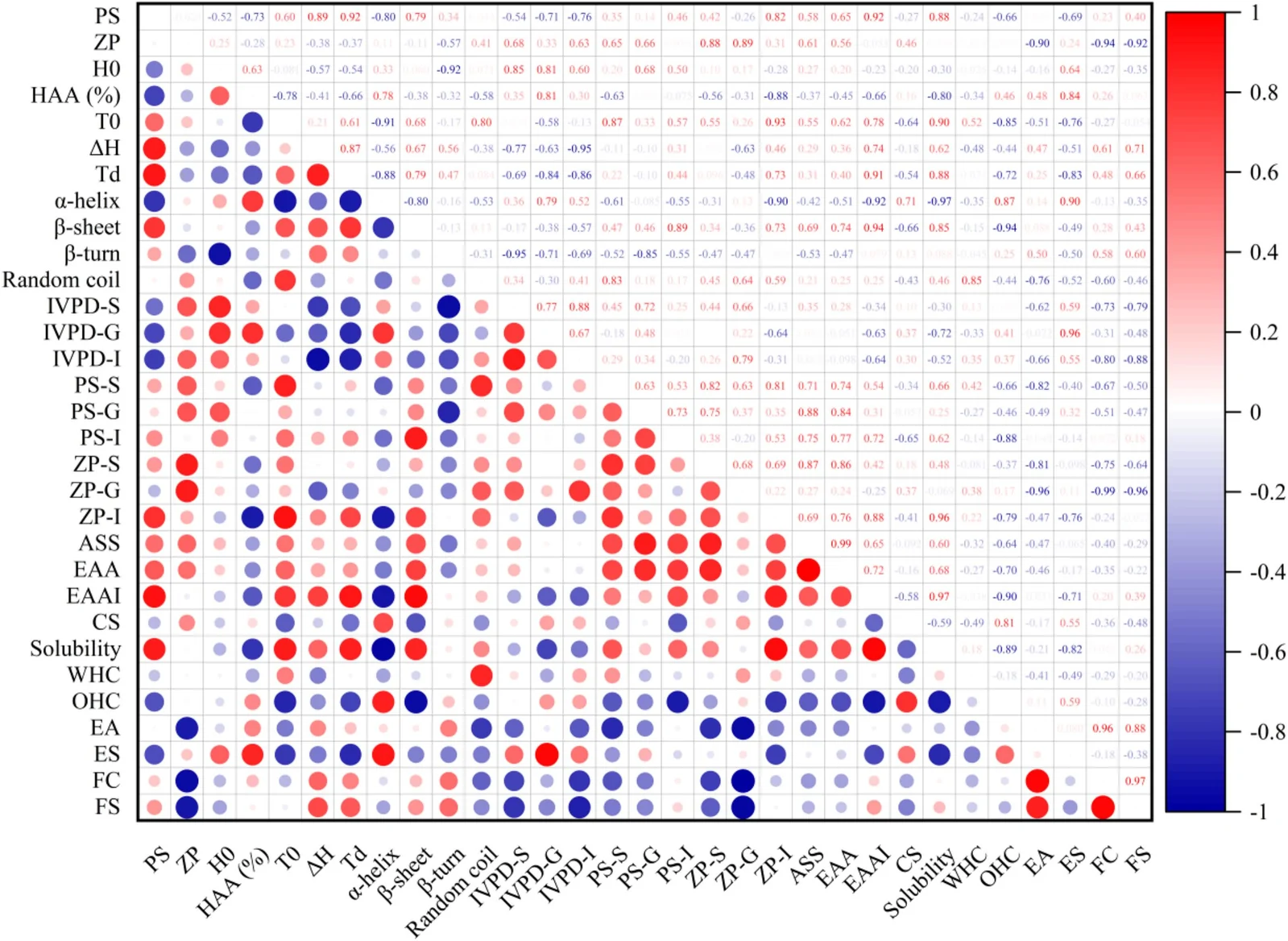
Pearson correlation matrix heatmap of the structural, physical, nutritional, and functional properties of PSP. The color scale on the right represents the Pearson correlation coefficient (r), ranging from −1 (blue, negative correlation) to 1 (red, positive correlation). The values in the cells indicate specific correlation coefficients. (For interpretation of the references to color in this figure legend, the reader is referred to the web version of this article.)

Particle size, solubility, and conformational flexibility were closely related to digestibility. IVPD was positively correlated with random coil content (*r* = 0.74) and solubility (*r* = 0.82) but negatively correlated with particle size (*r* = −0.76). These results indicated that well-dispersed and flexible protein structures were more accessible to digestive enzymes. This might explain the relatively high digestibility of PRO despite its low solubility and the reduced intestinal digestibility of GLU, which was likely related to secondary aggregation under intestinal conditions.

Surface hydrophobicity was strongly associated with oil-binding and emulsion-related properties. H₀ showed positive correlations with OHC and ESI, with the strongest correlation reaching *r* = 0.85, while it was negatively correlated with solubility and WHC (*r* = −0.87 to −0.89). These results indicated that exposed hydrophobic groups promoted lipid interaction and interfacial stabilization but weakened water affinity. Previous studies have similarly shown that surface hydrophobicity and interfacial adsorption were key factors governing the emulsifying behavior of plant proteins (Eskandari et al., 2025; Lima et al., 2024). Nutritional indices, including EAA, AAS, EAAI, and CS, were also strongly correlated with each other, with correlation coefficients up to *r* = 0.91, reflecting their common dependence on amino acid composition.

The relationships among structural, physicochemical, nutritional, and functional indicators of PSP fractions were summarized by the correlation analysis. Ordered secondary structures were mainly associated with thermal stability, whereas particle size, solubility, and conformational flexibility were linked to digestive accessibility. Surface hydrophobicity was closely related to oil-binding capacity and emulsion stability, while amino acid composition was reflected in the nutritional indices. These correlations provided an integrated view of how fraction-specific structural characteristics were connected with functional and nutritional performance, and a systematic basis was offered for understanding the structure–function relationship of PSP fractions.

## 4 Conclusion

This study demonstrated that PSP is a compositionally heterogeneous protein source and that alkaline–isoelectric precipitation combined with Osborne fractionation can separate PSP into fractions with distinct structural, functional, nutritional, and digestive characteristics. Structural analyses showed that differences in particle size, surface hydrophobicity, secondary structure, colloidal stability, and thermal behavior were closely associated with fraction-specific protein composition. From a nutritional perspective, ALB exhibited the most favorable overall profile, whereas PRO showed complementary potential because of its high intestinal digestibility and low phytic acid content, although its unbalanced amino acid composition remains a limitation. DIA-based proteomic profiling revealed fraction-specific enrichment of storage, lipid-transfer-related, metabolic, and protein-homeostasis-associated proteins, providing complementary compositional information for interpreting the differences among PSP fractions. Further functional validation was needed to confirm their specific contributions to the observed properties. These findings provide a molecular and structural basis for the potential targeted utilization of PSP fractions as sustainable plant-based protein ingredients. These fraction-specific characteristics, combined with the utilization of pomegranate seeds as an underused juice-processing by-product, suggested that PSP fractions might have provided complementary options for selected food applications. Proteomic screening also identified several proteins with previously reported IgE-binding activity, indicating that targeted immunological assessment is required before the PSP fractions are considered for food applications. Further studies should therefore evaluate the performance of individual PSP fractions in model and practical food systems and improve their limiting amino acid profiles and digestibility to expand their potential food applications.

## CRediT authorship contribution statement

**Lin Yuan:** Writing – original draft, Visualization, Methodology, Investigation, Formal analysis, Data curation. **Yu Qiu:** Methodology, Formal analysis. **Jiayan Yang:** Visualization, Software. **Qianyang Fang:** Visualization, Data curation. **Qianlin Deng:** Software, Resources. **Jingyang Hong:** Project administration, Investigation. **Mengmeng Zhao:** Writing – review & editing, Validation, Resources, Funding acquisition. **Min Zhu:** Writing – review & editing, Supervision, Resources, Funding acquisition, Conceptualization.

## Declaration of competing interest

The authors declare that they have no known competing financial interests or personal relationships that could have appeared to influence the work reported in this paper.

## Data Availability

Data will be made available on request.

## Appendix A Supplementary data

Supplementary material SDS-PAGE, FTIR secondary-structure analysis, DSC thermograms, protein extraction yield, purity and recovery, differentially abundant proteins, and potential allergenic proteins of pomegranate seed protein fractions.
