# Supplementary material for: Osborne fractionation of pomegranate seed protein: Structural characteristics, processing properties, proteomic profiles and nutritional value
Source: Food Chem X. 2026 Oct 3;39:104551. doi: 10.1016/j.fochx.2026.104551 (PMC13671052; doi:10.1016/j.fochx.2026.104551)
Supplement: Supplementary file 1 [file mmc1.docx]

**Supplementary material**

## Osborne fractionation of pomegranate seed protein: Structural characteristics, processing properties, proteomic profiles and nutritional value

Lin Yuan ^a^, Yu Qiu ^a^, Jiayan Yang ^a^, Qianyang Fang ^a^, Qianlin Deng ^a^, Jingyang Hong ^a, b^, Mengmeng Zhao ^a, b,^ *, Min Zhu ^a, b,^ *

^a^ College of Smart Agriculture (Research Institute), Xinjiang University, Urumqi, 830046, China

^b^ Xinjiang Silk Research Institute（Fruit and Vegetable Processing), Urumqi, 830046, China

* Correspondence: [zmengmeng0805@126.com;](mailto:zmengmeng0805@126.com;) [minna9071@126.com](mailto:minna9071@126.com)

**Figure caption**

**Fig. S1.** SDS-PAGE profiles of different PSP fractions under (A) reducing conditions and (B) non-reducing conditions.

**Fig. S2** Deconvolution and second-derivative fitting results of FTIR amide I band (1700–1600 cm⁻¹) for different PSP fractions.

**Fig. S3.** DSC thermograms of different PSP fractions, (A) ALB, (B) GLO, (C) PRO, (D) GLU, and (E) CP. The corresponding thermal parameters were indicated on the thermograms and summarized in Table 1 of the manuscript.

**Fig. S1**

**Fig. S2**

**Fig. S3**

**Table S1.**

Differentially abundant proteins identified of different PSP fractions.

| Rank | Accession | Description | ALB-Mean | GLO-Mean | PRO-Mean | GLU-Mean | CP-Mean | F-value | P-value | Functional Annotation |
| --- | --- | --- | --- | --- | --- | --- | --- | --- | --- | --- |
| 1 | A0A218WFE9 | Nucleolin-like | 2624.76 | 41.25 | 41.25 | 41.25 | 41.25 | 7002.91 | 1.357×10⁻¹³ | Nucleolar protein involved in ribosome biogenesis and RNA processing |
| 2 | A0A218X2K8 | Nuclear pore protein | 41.25 | 41.25 | 41.25 | 41.25 | 9550.34 | 5690.35 | 1.914×10⁻¹³ | Nuclear pore complex, nucleocytoplasmic transport |
| 3 | A0A218XT20 | ABC transporter I family member 6, chloroplastic | 41.25 | 41.25 | 41.25 | 41.25 | 14664.87 | 4245.69 | 5.514×10⁻¹³ | Chloroplast ABC transporter |
| 4 | A0A218WE44 | small monomeric GTPase | 41.25 | 41.25 | 41.25 | 41.25 | 887.52 | 1862.39 | 2.535×10⁻¹¹ | Signal transduction |
| 5 | A0A218W5M0 | Protein VAC14 homolog | 41.25 | 41.25 | 41.25 | 41.25 | 25911.9 | 1437.01 | 7.398×10⁻¹¹ | Vacuolar protein sorting |
| 6 | A0A6P8E4K8 | Protein GRIP | 80987.7 | 41.25 | 41.25 | 41.25 | 142738.67 | 1374.87 | 7.687×10⁻¹¹ | Golgi-associated protein |
| 7 | A0A218X6H3 | Glyceraldehyd× 103-phosphate dehydrogenase (GAPDH) | 7304.86 | 41.25 | 41.25 | 41.25 | 3973.61 | 1284.41 | 9.253×10⁻¹¹ | Key enzyme in glycolysis |
| 8 | A0A6P8BSM8 | acetyl-CoA C-acyltransferase | 22495.9 | 41.25 | 41.25 | 41.25 | 60691.5 | 1201.86 | 1.128×10⁻¹⁰ | Fatty acid metabolism |
| 9 | A0A218XS85 | Uricase | 41.25 | 41.25 | 41.25 | 204393 | 9876.81 | 1074.04 | 1.756×10⁻¹⁰ | Purine metabolism |
| 10 | A0A218XW29 | BURP domain-containing protein BNM2A-like | 41.25 | 41.25 | 41.25 | 41.25 | 10556.7 | 993.58 | 2.119×10⁻¹⁰ | Seed development and stress response |
| 11 | A0A6P8DZH9 | Vacuolar protein sorting-associated protein 52 A | 41.25 | 41.25 | 41.25 | 41.25 | 132244.67 | 1004.69 | 2.12×10^-10^ | vacuolar protein sorting-associated protein 52 A |
| 12 | A0A218WB81 | Protein EARLY RESPONSIVE TO DEHYDRATION 15-like | 781.07 | 41.25 | 41.25 | 41.25 | 755.27 | 889.57 | 3.37×10^-10^ | protein EARLY RESPONSIVE TO DEHYDRATION 15-like |
| 13 | A0A218VY22 | cysteine dioxygenase | 608.65 | 41.25 | 41.25 | 41.25 | 41.25 | 867 | 3.54×10-^10^ | plant cysteine oxidase 3 isoform X1 |
| 14 | A0A6P8C9P6 | DDRGK domain-containing protein 1 | 41.25 | 41.25 | 41.25 | 41.25 | 97991.4 | 843.24 | 3.77×10-^10^ | DDRGK domain-containing protein 1 |
| 15 | A0A218W712 | Uncharacterized protein LOC116194616 | 41.25 | 41.25 | 41.25 | 41.25 | 273716 | 802.34 | 4.46×10-^10^ | uncharacterized protein LOC116194616 |
| 16 | A0A218X9Y5 | Structural maintenance of chromosomes protein | 41.25 | 41.25 | 41.25 | 41.25 | 156775.33 | 793.58 | 4.46×10-^10^ | hypothetical protein CDL15-Pgr007534 |
| 17 | A0A2I0I436 | Alpha-1,4 glucan phosphorylase (Fragment) | 17974.87 | 41.25 | 41.25 | 41.25 | 49818.2 | 767.36 | 4.97×10-^10^ | hypothetical protein CRG98-040857, partial |
| 18 | A0A218WJE3 | Long-chain-fatty-acid--CoA ligase | 238569.33 | 41.25 | 41.25 | 41.25 | 86347.43 | 733.08 | 5.58×10-^10^ | long chain acyl-CoA synthetase 4 |
| 19 | A0A218XVR5 | Chaperone protein dnaJ 13 | 41.25 | 41.25 | 41.25 | 41.25 | 10012.63 | 739.05 | 5.58×10-^10^ | chaperone protein dnaJ 13 |
| 20 | A0A218XV21 | NADH dehydrogenase [ubiquinone] 1 beta subcomplex subunit 8, mitochondrial | 41.25 | 41.25 | 41.25 | 41.25 | 55261.83 | 721.23 | 5.75×10-^10^ | NADH dehydrogenase [ubiquinone] 1 beta subcomplex subunit 8, mitochondrial |
| 21 | A0A218VRP6 | PX domain-containing protein | 41.25 | 41.25 | 41.25 | 41.25 | 569.08 | 706.92 | 6.05×10-^10^ | hypothetical protein CDL15-Pgr026197 |
| 22 | A0A218W2U4 | Late embryogenesis abundant protein D-34-like | 1602650 | 112805 | 660006.67 | 41.25 | 73980.2 | 656.06 | 8.38×10-^10^ | late embryogenesis abundant protein D-34-like |
| 23 | A0A218XPP4 | Singl× 10stranded DNA-binding protein WHY2, mitochondrial | 1117.71 | 41.25 | 41.25 | 41.25 | 1062.74 | 611.03 | 1.14×10-^9^ | single-stranded DNA-binding protein WHY2, mitochondrial |
| 24 | A0A218XA33 | Myosin-9 | 1023982 | 455456.67 | 927197.67 | 41.25 | 2645146.67 | 584.77 | 1.36×10-^9^ | myosin-9 |
| 25 | A0A218WYJ1 | Succinate dehydrogenase assembly factor 2, mitochondrial | 118328.33 | 41.25 | 41.25 | 41.25 | 260840.67 | 565.44 | 1.47×10-^9^ | succinate dehydrogenase assembly factor 2, mitochondrial |
| 26 | A0A218Y1Z4 | Eukaryotic translation initiation factor 3 subunit D | 120567.53 | 41.25 | 41.25 | 1345516.67 | 628370.67 | 569.29 | 1.47×10^-9^ | eukaryotic translation initiation factor 3 subunit D |
| 27 | A0A2I0LG00 | glucos× 106-phosphate 1-epimerase | 49081.53 | 661635.33 | 68243 | 41.25 | 75337.7 | 562.12 | 1.47×10^-9^ | putative glucose-6-phosphate 1-epimerase |
| 28 | A0A218XLH6 | Uncharacterized protein | 41.25 | 41.25 | 41.25 | 41.25 | 12802.33 | 548.33 | 1.61×10^-9^ | hypothetical protein CDL15-Pgr012063 |
| 29 | A0A218WD19 | Syntaxin-31 | 509.13 | 41.25 | 41.25 | 41.25 | 41.25 | 536.46 | 1.73×10^-9^ | syntaxin-31 |
| 30 | A0A218X2X9 | Spidroin-1-like | 111482666.7 | 9350793.33 | 86284900 | 3391720 | 67502266.67 | 514.33 | 2.06×10^-9^ | spidroin-1-like |
| 31 | A0A218WBA5 | ATP synthase subunit d, mitochondrial | 575174.33 | 585008.33 | 1540653.33 | 635197.67 | 3983846.67 | 488.74 | 2.34×10^-9^ | ATP synthase subunit d, mitochondrial |
| 32 | A0A218WZS4 | D-glycerate 3-kinase, chloroplastic | 3270.58 | 41.25 | 41.25 | 41.25 | 5192.18 | 495.72 | 2.34×10^-9^ | D-glycerate 3-kinase, chloroplastic |
| 33 | A0A218XEW9 | Uncharacterized protein | 41.25 | 41.25 | 41.25 | 41.25 | 110810 | 490.62 | 2.34×10^-9^ | hypothetical protein CDL15-Pgr012757 |
| 34 | A0A2I0J9I6 | Eukaryotic translation initiation factor 4B3-like | 744228.33 | 41.25 | 540356 | 41.25 | 378713.33 | 490.7 | 2.34×10^-9^ | hypothetical protein CRG98-026738 |
| 35 | A0A218WBB1 | 60S ribosomal protein L34 | 10589.92 | 41.25 | 41.25 | 586675.33 | 30674.27 | 466.33 | 2.80×10^-9^ | 60S ribosomal protein L34 |
| 36 | A0A218XIC8 | Protein CROWDED NUCLEI 1 | 53480.03 | 41.25 | 66772.17 | 41.25 | 78955.53 | 467.97 | 2.80×10^-9^ | hypothetical protein CDL15-Pgr027461 |
| 37 | A0A218X7E4 | CTLH domain-containing protein | 41.25 | 41.25 | 41.25 | 41.25 | 5034 | 438.93 | 3.37×10^-9^ | hypothetical protein CDL15-Pgr006915 |
| 38 | A0A218XCD8 | AMP-activated protein kinase glycogen-binding domain-containing protein | 52676.73 | 41.25 | 41.25 | 41.25 | 277054.33 | 443.9 | 3.37×10^-9^ | hypothetical protein CDL15-Pgr002012 |
| 39 | A0A218XCG8 | Embryonic protein DC-8-like | 51065900 | 3641600 | 33241566.67 | 3019020 | 43141233.33 | 435.79 | 3.37×10^-9^ | embryonic protein DC-8-like |
| 40 | A0A218XUT9 | Dehydrin Rab18-like | 610-98566.67 | 7133183.33 | 67628600 | 829069.33 | 6268993.33 | 445.65 | 3.37×10^-9^ | dehydrin Rab18-like |
| 41 | A0A218XVM7 | UspA domain-containing protein | 267394.67 | 41.25 | 15420.7 | 41.25 | 647038 | 435.43 | 3.37×10^-9^ | hypothetical protein CDL15-Pgr002463 |
| 42 | A0A2I0JV23 | Serine/threonin× 10protein phosphatase | 41.25 | 41.25 | 41.25 | 41.25 | 3771.94 | 440.8 | 3.37×10^-9^ | hypothetical protein CISIN-1g022085mg |
| 43 | A0A2I0HN24 | 3'(2'),5'-bisphosphate nucleotidase | 4355580 | 41.25 | 41.25 | 41.25 | 4384253.33 | 419.29 | 3.97×10^-9^ | hypothetical protein CRG98-046759 |
| 44 | A0A218VRW8 | Prostaglandin E synthase 2 | 41.25 | 41.25 | 41.25 | 41.25 | 57171.5 | 412.42 | 4.08×10^-9^ | hypothetical protein CDL15-Pgr019936 |
| 45 | A0A218WCW1 | Uncharacterized protein | 11551.77 | 41.25 | 41.25 | 41.25 | 12513.33 | 411.32 | 4.08×10^-9^ | hypothetical protein CDL15-Pgr014367 |
| 46 | A0A218XKN4 | Signal peptidase complex subunit 3 | 237.71 | 41.25 | 41.25 | 41.25 | 6958.3 | 413.76 | 4.08×10^-9^ | hypothetical protein CDL15-Pgr019002 |
| 47 | A0A2I0J7Y4 | Uncharacterized protein LOC116200103 | 41.25 | 41.25 | 41.25 | 41.25 | 7751.37 | 403.8 | 4.38×10^-9^ | uncharacterized protein LOC116200103 |
| 48 | A0A218XQS0 | Cysteine proteinase inhibitor | 5648960 | 1773106 | 3220903.33 | 41.25 | 19866966.67 | 399.95 | 4.50×10^-9^ | hypothetical protein CDL15-Pgr022391 |
| 49 | A0A218VUI8 | Abscisic stress-ripening protein 1-like | 1043429.33 | 3840473.33 | 3813940 | 1004068.67 | 25114566.67 | 392.39 | 4.74×10^-9^ | abscisic stress-ripening protein 1-like |
| 50 | A0A218X854 | Myosin heavy chain, clone 203 | 183580.33 | 41.25 | 272123.33 | 41.25 | 229128.33 | 393.93 | 4.74×10^-9^ | myosin heavy chain, clone 203 |
| 51 | A0A218W2M6 | Uncharacterized protein | 41.25 | 41.25 | 41.25 | 41.25 | 38487.9 | 386.79 | 5.00×10^-9^ | hypothetical protein CDL15-Pgr010432 |
| 52 | A0A218WFA3 | 28 kDa ribonucleoprotein, chloroplastic-like | 548683 | 373079 | 609040 | 41.25 | 2223520 | 385.17 | 5.00×10^-9^ | 28 kDa ribonucleoprotein, chloroplastic-like |
| 53 | A0A2I0I9J4 | Gamma carbonic anhydrase 1, mitochondrial-like | 41.25 | 41.25 | 41.25 | 41.25 | 22729.03 | 383.43 | 5.02×10^-9^ | hypothetical protein CRG98-039566 |
| 54 | A0A218VVH5 | RING-type E3 ubiquitin transferase | 37401 | 41.25 | 70919.17 | 41.25 | 165968.67 | 380.27 | 5.13×10^-9^ | hypothetical protein CDL15-Pgr020514 |
| 55 | A0A2I0I7V3 | NADH dehydrogenase [ubiquinone] 1 alpha subcomplex subunit 12 | 83846.23 | 41.25 | 140803.67 | 41.25 | 211580.33 | 378.77 | 5.14×10^-9^ | probable NADH dehydrogenase [ubiquinone] 1 alpha subcomplex subunit 12 isoform X1 |
| 56 | A0A218WCB2 | Aminomethyltransferase | 19414.3 | 41.25 | 98970.37 | 41.25 | 14850.97 | 369.43 | 5.65×10^-9^ | aminomethyltransferase, mitochondrial |
| 57 | A0A218WWL5 | threonine synthase | 69746.43 | 41.25 | 41.25 | 41.25 | 33316.07 | 368.6 | 5.65×10^-9^ | threonine synthase, chloroplastic-like |
| 58 | A0A2I0KKF8 | Uncharacterized protein LOC116206185 | 214138 | 340669 | 528716 | 41.25 | 1786950 | 367.62 | 5.65×10^-9^ | uncharacterized protein LOC116206185 |
| 59 | A0A2I0HP54 | TFIIS N-terminal domain-containing protein | 2158.98 | 41.25 | 41.25 | 41.25 | 41.25 | 363.36 | 5.89×10^-9^ | hypothetical protein CRG98-046354 |
| 60 | A0A218XU67 | Exocyst complex component SEC10b | 3295.84 | 41.25 | 41.25 | 41.25 | 81282.47 | 360.05 | 6.06×10^-9^ | exocyst complex component SEC10b |
| 61 | A0A218VWS1 | 14-3-3-like protein A | 7320736.67 | 2035796.67 | 2369093.33 | 1453933.33 | 12196166.67 | 353.76 | 6.40×10^-9^ | 14-3-3-like protein A |
| 62 | A0A6P8E808 | Cytochrome c oxidase subunit 5b-1, mitochondrial-like | 366494.33 | 359251 | 430627 | 41.25 | 2731810 | 354.43 | 6.40×10^-9^ | cytochrome c oxidase subunit 5b-1, mitochondrial-like |
| 63 | A0A218X2S4 | ENT domain-containing protein | 230163.33 | 41.25 | 41.25 | 41.25 | 499881.67 | 350.54 | 6.46×10^-9^ | hypothetical protein CDL15-Pgr003131 |
| 64 | A0A218X666 | WD-40 repeat-containing protein MSI3-like | 8354.58 | 41.25 | 41.25 | 41.25 | 97694.77 | 351.67 | 6.46×10^-9^ | WD-40 repeat-containing protein MSI3-like |
| 65 | A0A218XIC6 | Uncharacterized protein LOC116202608 | 27393.53 | 41.25 | 41.25 | 41.25 | 145554.33 | 349.68 | 6.46×10^-9^ | uncharacterized protein LOC116202608 |
| 66 | A0A218VW84 | ABC transporter F family member 4 | 789714.67 | 41.25 | 719575.67 | 41.25 | 389924.33 | 347.71 | 6.55×10^-9^ | ABC transporter F family member 4 |
| 67 | A0A218XY95 | Protein NLRC3 isoform X1 | 41.25 | 41.25 | 41.25 | 41.25 | 138788.67 | 344.69 | 6.64×10^-9^ | protein NLRC3 isoform X1 |
| 68 | A0A2I0JHS0 | Presequence protease 2, chloroplastic/mitochondrial | 80510.2 | 41.25 | 41.25 | 41.25 | 218843.67 | 344.85 | 6.64×10^-9^ | presequence protease 2, chloroplastic/mitochondrial |
| 69 | A0A218WQY6 | Methylthioribos× 101-phosphate isomerase | 77631.3 | 41.25 | 41.25 | 41.25 | 191078.33 | 340.48 | 6.85×10^-9^ | methylthioribose-1-phosphate isomerase |
| 70 | A0A218X122 | Translation initiation factor eIF2B subunit beta | 12285.57 | 41.25 | 41.25 | 41.25 | 35635 | 341.13 | 6.85×10^-9^ | translation initiation factor eIF-2B subunit beta |
| 71 | A0A218VYS5 | Iron-sulfur cluster assembly protein | 57068.37 | 41.25 | 41.25 | 41.25 | 164519.67 | 338.77 | 6.93×10^-9^ | iron-sulfur cluster assembly protein 1-like |
| 72 | A0A218WFX5 | Outer envelope pore protein 16-2, chloroplastic | 3402480 | 5807410 | 8040930 | 1543686.67 | 24197000 | 334.34 | 7.29×10^-9^ | outer envelope pore protein 16-2, chloroplastic |
| 73 | A0A2I0L829 | Glutaredoxin-dependent peroxiredoxin | 16024833.33 | 1910053.33 | 2201253.33 | 1506566.67 | 7872866.67 | 330.53 | 7.61×10^-9^ | peroxiredoxin-2 |
| 74 | A0A218W8K5 | Protein transport protein Sec61 subunit beta | 41.25 | 41.25 | 41.25 | 41.25 | 882388.33 | 329.55 | 7.62×10^-9^ | protein transport protein Sec61 subunit beta |
| 75 | A0A218WPE0 | Sucrose synthase | 2102.9 | 41.25 | 41.25 | 57487.57 | 7124.36 | 327.22 | 7.79×10^-9^ | sucrose synthase |
| 76 | A0A218WEC5 | Succinate dehydrogenase subunit 5, mitochondrial | 705598 | 276487.53 | 649186.67 | 45100.5 | 2977293.33 | 320.69 | 8.07×10^-9^ | hypothetical protein CDL15-Pgr014566 |
| 77 | A0A218WJ70 | Sister chromatid cohesion 1 protein 4 | 82279.13 | 41.25 | 41.25 | 41.25 | 194461.33 | 322.11 | 8.07×10^-9^ | hypothetical protein CDL15-Pgr018176 |
| 78 | A0A218XKK9 | non-specific serine/threonine protein kinase | 27980.5 | 41.25 | 41.25 | 41.25 | 110175.67 | 321.24 | 8.07×10^-9^ | serine/threonine-protein kinase BSK7-like isoform X2 |
| 79 | A0A2I0ILP6 | Ubiquitin-40S ribosomal protein S27a | 3315.26 | 41.25 | 41.25 | 104329.27 | 1289.84 | 321.25 | 8.07×10^-9^ | ubiquitin-40S ribosomal protein S27a |
| 80 | A0A2I0KAT0 | Uncharacterized protein | 41.25 | 41.25 | 41.25 | 41.25 | 15455.9 | 321.9 | 8.07×10^-9^ | hypothetical protein CRG98-013927 |
| 81 | A0A218X951 | Malate dehydrogenase | 5219806.67 | 6011553.33 | 1393113.33 | 310219 | 1577136.67 | 316.21 | 8.42×10^-9^ | malate dehydrogenase, mitochondrial |
| 82 | A0A2I0JR45 | Aconitase/3-isopropylmalate dehydratase large subunit alpha/beta/alpha domain-containing protein | 28782.6 | 41.25 | 41.25 | 41.25 | 32575.83 | 316.78 | 8.42×10^-9^ | 3-isopropylmalate dehydratase large subunit, chloroplastic |
| 83 | A0A2I0JYQ5 | cinnamyl-alcohol dehydrogenase | 8358.83 | 41.25 | 41.25 | 41.25 | 8886.96 | 315.55 | 8.42×10^-9^ | hypothetical protein CRG98-018141 |
| 84 | A0A218WKZ4 | Serine/threonin× 10protein phosphatase 5 | 43151.9 | 41.25 | 41.25 | 41.25 | 57994.13 | 314.27 | 8.43×10^-9^ | serine/threonine-protein phosphatase 5 isoform X1 |
| 85 | A0A218X4L3 | Small ribosomal subunit protein uS2 | 78748.67 | 41.25 | 101034.5 | 41.25 | 358882.67 | 313.32 | 8.43×10^-9^ | 40S ribosomal protein SA-like isoform X2 |
| 86 | A0A2I0K615 | E2F-associated phosphoprotein isoform X1 | 41.25 | 41.25 | 41.25 | 41.25 | 319820 | 313.26 | 8.43×10^-9^ | E2F-associated phosphoprotein isoform X1 |
| 87 | A0A218WSR2 | Hypersensitiv× 10induced response protein 4 | 16234.95 | 41.25 | 106092.67 | 41.25 | 54068.5 | 308.06 | 8.95×10^-9^ | hypersensitive-induced response protein 4 |
| 88 | A0A218Y2M3 | 60S ribosomal protein L38 | 152423.67 | 41.25 | 41.25 | 41.25 | 189610.33 | 308.24 | 8.95×10^-9^ | 60S ribosomal protein L38 |
| 89 | A0A218WAC6 | Importin subunit alpha | 15151.17 | 41.25 | 41.25 | 41.25 | 79213.13 | 304.14 | 9.43×10^-9^ | hypothetical protein CDL15-Pgr025584 |
| 90 | A0A218XLJ9 | Large ribosomal subunit protein eL14 domain-containing protein | 46485.73 | 22163.17 | 98672.93 | 492648.67 | 85572.73 | 302.46 | 9.58×10^-9^ | hypothetical protein CDL15-Pgr023707 |
| 91 | A0A218W9U8 | Late embryogenesis abundant protein ECP63-like | 4171256.67 | 669580.33 | 1939226.67 | 41.25 | 775002 | 299.63 | 9.93×10^-9^ | late embryogenesis abundant protein ECP63-like |
| 92 | A0A218XQ55 | Two-on-two hemoglobin-3 | 43219.57 | 41.25 | 41.25 | 41.25 | 137392.33 | 298.33 | 1.00×10^-8^ | two-on-two hemoglobin-3 |
| 93 | A0A218X140 | Bet v I/Major latex protein domain-containing protein | 5401070 | 694792 | 745102.67 | 41.25 | 1502540 | 297.48 | 1.01×10^-8^ | hypothetical protein CDL15-Pgr002824 |
| 94 | A0A218VUR1 | FT-interacting protein 3-like | 6394.49 | 41.25 | 41.25 | 41.25 | 5019.2 | 294.9 | 1.03×10^-8^ | FT-interacting protein 3-like |
| 95 | A0A2I0J1X6 | Probable 2-oxoglutarat× 10dependent dioxygenase At5g05600 | 76393.43 | 41.25 | 41.25 | 41.25 | 62392.57 | 294.84 | 1.03×10^-8^ | probable 2-oxoglutarate-dependent dioxygenase At5g05600 |
| 96 | A0A2I0LFF4 | Eukaryotic translation initiation factor 5A | 234054.33 | 41.25 | 258506 | 41.25 | 524438.67 | 294.16 | 1.03×10^-8^ | eukaryotic translation initiation factor 5A-like |
| 97 | A0A2I0IJE1 | Uncharacterized protein LOC116209878 | 52156.87 | 41.25 | 41.25 | 41.25 | 27026.33 | 290.86 | 1.08×10^-8^ | uncharacterized protein LOC116209878 |
| 98 | A0A218WP29 | Vacuolar protein sorting-associated protein 24 homolog 1 | 41.25 | 41.25 | 41.25 | 41.25 | 1089.6 | 277.35 | 1.35×10^-8^ | vacuolar protein sorting-associated protein 24 homolog 1 |
| 99 | A0A218VXR9 | Endoplasmic reticulum membrane protein C16E8.02-like | 162.1 | 41.25 | 41.25 | 41.25 | 2171.29 | 274.32 | 1.41×10^-8^ | hypothetical protein CDL15-Pgr008875 |
| 100 | A0A218WHA9 | Transferase At4g12130, mitochondrial | 207097.33 | 11584.77 | 94418.07 | 41.25 | 265654.33 | 273.8 | 1.41×10^-8^ | putative transferase At4g12130, mitochondrial |
| 101 | A0A218WXZ1 | Uncharacterized protein | 418666.67 | 288205 | 533352.67 | 41.25 | 969923.33 | 272.26 | 1.44×10^-8^ | hypothetical protein CDL15-Pgr028887 |
| 102 | A0A218VV21 | Dolichyl-diphosphooligosaccharid× 10-protein glycosyltransferase subunit DAD1 | 41.25 | 41.25 | 41.25 | 41.25 | 4081.22 | 271.46 | 1.44×10^-8^ | dolichyl-diphosphooligosaccharide--protein glycosyltransferase subunit DAD1 |
| 103 | A0A218XJ92 | Glutathione S-transferase DHAR3, chloroplastic | 5488136.67 | 1422213.33 | 943234 | 41.25 | 3678906.67 | 270.47 | 1.44×10^-8^ | glutathione S-transferase DHAR3, chloroplastic |
| 104 | A0A6P8CW30 | Nucleosome assembly protein 13-like isoform X2 | 41.25 | 41.25 | 41.25 | 41.25 | 571432 | 270.76 | 1.44×10^-8^ | nucleosome assembly protein 1;3-like isoform X2 |
| 105 | A0A218XMG0 | Protein transport protein sec16 | 41.25 | 41.25 | 41.25 | 41.25 | 6251.64 | 269.77 | 1.45×10^-8^ | hypothetical protein CDL15-Pgr021522 |
| 106 | A0A1Z1GAK6 | ATP-dependent Clp protease proteolytic subunit | 19289.6 | 41.25 | 41.25 | 41.25 | 15514.9 | 264.87 | 1.57×10^-8^ | clp protease proteolytic subunit |
| 107 | A0A218VWH2 | transketolase | 182465.67 | 41.25 | 208038 | 41.25 | 248669.67 | 259.23 | 1.70×10^-8^ | transketolase, chloroplastic |
| 108 | A0A218WB75 | Small ribosomal subunit protein uS15c | 20393.83 | 41.25 | 41.25 | 41.25 | 82091.53 | 258.22 | 1.70×10^-8^ | uncharacterized protein LOC116199706 isoform X2 |
| 10-9 | A0A218WIM2 | Starch synthase, chloroplastic/amyloplastic | 41.25 | 41.25 | 41.25 | 37614.63 | 41.25 | 258.19 | 1.70×10^-8^ | granule-bound starch synthase 1, chloroplastic/amyloplastic-like |
| 110 | A0A218WZX6 | BSD domain-containing protein 1 | 14228.67 | 41.25 | 41.25 | 41.25 | 26140.1 | 258.14 | 1.70×10^-8^ | BSD domain-containing protein 1 |
| 111 | A0A218XIJ6 | Uncharacterized protein LOC116196080 | 41.25 | 41.25 | 41.25 | 41.25 | 115178.33 | 259.34 | 1.70×10^-8^ | uncharacterized protein LOC116196080 |
| 112 | A0A218WNK4 | non-specific serine/threonine protein kinase | 9100.45 | 41.25 | 41.25 | 41.25 | 9908.21 | 257.04 | 1.72×10^-8^ | CBL-interacting serine/threonine-protein kinase 3-like isoform X1 |
| 113 | A0A6P8CAU8 | UMP-CMP kinase | 136539 | 41.25 | 41.25 | 41.25 | 161614 | 253.88 | 1.81×10^-8^ | UMP-CMP kinase 4-like |
| 114 | A0A218XJA4 | Cleavage and polyadenylation specificity factor subunit 3-I | 41.25 | 41.25 | 41.25 | 41.25 | 12485.83 | 251.56 | 1.86×10^-8^ | hypothetical protein CDL15-Pgr027675 |
| 115 | A0A6P8C826 | Nuclear-pore anchor | 291751 | 290538.67 | 266900.67 | 41.25 | 451220 | 252.25 | 1.86×10^-8^ | nuclear-pore anchor |
| 116 | A0A6P8CPS6 | NEDD8 ultimate buster 1 | 207062.33 | 41.25 | 186036.33 | 41.25 | 295297.67 | 251.39 | 1.86×10^-8^ | NEDD8 ultimate buster 1 |
| 117 | A0A2I0IGD4 | Neurofilament heavy polypeptid× 10like | 608249 | 41.25 | 604865 | 41.25 | 237893 | 249.23 | 1.92×10^-8^ | neurofilament heavy polypeptide-like |
| 118 | A0A218W358 | Cinnamoyl-CoA reductase 1-like | 16802.3 | 41.25 | 41.25 | 41.25 | 34746.7 | 245.39 | 1.97×10^-8^ | cinnamoyl-CoA reductase 1-like |
| 119 | A0A218X907 | Uncharacterized protein | 125566.67 | 41.25 | 96442.07 | 41.25 | 19558.9 | 247.19 | 1.97×10^-8^ | hypothetical protein CDL15-Pgr007221 |
| 120 | A0A218XYY0 | Uncharacterized protein At4g37920 isoform X1 | 41.25 | 41.25 | 41.25 | 41.25 | 526.43 | 245.63 | 1.97×10^-8^ | uncharacterized protein At4g37920 isoform X1 |
| 121 | A0A218XZ08 | Transcription factor Pur-alpha 1 | 85645 | 41.25 | 96426.73 | 41.25 | 140872 | 246.48 | 1.97×10^-8^ | transcription factor Pur-alpha 1 |
| 122 | A0A218XZE0 | Oleosin 1-like | 616311.67 | 621346.33 | 925779.67 | 111707.5 | 5601416.67 | 246.9 | 1.97×10^-8^ | oleosin 1-like |
| 123 | A0A2I0JVQ8 | Probable calcium-binding protein CML13 | 807885.33 | 620407 | 607756.67 | 41.25 | 4988803.33 | 245.49 | 1.97×10^-8^ | probable calcium-binding protein CML13 |
| 124 | A0A218VRS2 | Coatomer subunit zeta | 237191.67 | 41.25 | 41.25 | 41.25 | 320475.67 | 242.8 | 2.05×10^-8^ | hypothetical protein CDL15-Pgr026227 |
| 125 | A0A218WR01 | RRM domain-containing protein | 207318.67 | 41.25 | 438343 | 41.25 | 709532.67 | 242.51 | 2.05×10^-8^ | hypothetical protein CDL15-Pgr021280 |
| 126 | A0A218X8T7 | 3'(2'),5'-bisphosphate nucleotidase | 25280.2 | 41.25 | 41.25 | 41.25 | 13616.63 | 242.39 | 2.05×10^-8^ | PAP-specific phosphatase HAL2-like |
| 127 | A0A218WMV4 | 60S acidic ribosomal protein P2B-like | 1863473.33 | 741307.67 | 3658713.33 | 41.25 | 4790593.33 | 240.26 | 2.08×10^-8^ | hypothetical protein CDL15-Pgr026780 |
| 128 | A0A218X1Q1 | GrpE protein homolog | 1190713.33 | 256358.33 | 442314 | 41.25 | 1833246.67 | 240.9 | 2.08×10^-8^ | uncharacterized protein LOC116195955 isoform X1 |
| 129 | A0A218XRM8 | Exocyst subunit Exo70 family protein | 41.25 | 41.25 | 41.25 | 41.25 | 17168.6 | 241.07 | 2.08×10^-8^ | exocyst complex component EXO70A1-like |
| 130 | A0A218XX99 | 26S proteasome regulatory subunit RPN13 isoform X1 | 76704.33 | 41.25 | 41.25 | 41.25 | 206020 | 240.15 | 2.08×10^-8^ | 26S proteasome regulatory subunit RPN13 isoform X1 |
| 131 | A0A218XCB4 | Late embryogenesis abundant protein D-34-like | 29180066.67 | 2965230 | 16365166.67 | 224543.5 | 1303113.33 | 237.89 | 2.16×10^-8^ | late embryogenesis abundant protein D-34-like |
| 132 | A0A6P8C7I7 | Glutathione synthetase | 1424.98 | 41.25 | 980.4 | 20955.1 | 2203.93 | 237.48 | 2.16×10^-8^ | glutathione synthetase, chloroplastic |
| 133 | A0A218XAQ8 | Hsp70 nucleotide exchange factor fes1 | 1924780 | 253321.33 | 868198.33 | 87921.08 | 3295993.33 | 235.7 | 2.23×10^-8^ | hsp70 nucleotide exchange factor fes1 |
| 134 | A0A218XJA1 | fructokinase | 18321.7 | 41.25 | 41.25 | 41.25 | 62714.87 | 234.14 | 2.27×10^-8^ | probable fructokinase-6, chloroplastic |
| 135 | A0A6P8E0T3 | 15.7 kDa heat shock protein, peroxisomal | 324379.67 | 41.25 | 273859 | 41.25 | 222484 | 234.23 | 2.27×10^-8^ | 15.7 kDa heat shock protein, peroxisomal |
| 136 | A0A218XBB6 | Bifunctional aspartate aminotransferase and glutamate/aspartat× 10prephenate aminotransferase | 6131.84 | 41.25 | 41.25 | 41.25 | 5393.28 | 232.63 | 2.32×10^-8^ | hypothetical protein CDL15-Pgr001798 |
| 137 | A0A218XTZ7 | Uncharacterized protein LOC116204719 | 41.25 | 41.25 | 41.25 | 41.25 | 5497.6 | 229.26 | 2.46×10^-8^ | uncharacterized protein LOC116204719 |
| 138 | A0A218Y0C0 | 11S globulin subunit beta-like | 176358666.7 | 331903000 | 1502653333 | 2948560000 | 270772333.3 | 229.54 | 2.46×10^-8^ | 11S globulin subunit beta-like |
| 139 | A0A218WUX7 | Alpha-xylosidase 1 | 856566.33 | 1320973.33 | 1509960 | 300043.67 | 166345.33 | 228.4 | 2.47×10^-8^ | alpha-xylosidase 1 |
| 140 | A0A218XM10 | RNA helicase | 18588.33 | 41.25 | 35048.3 | 41.25 | 23079.87 | 228.6 | 2.47×10^-8^ | DExH-box ATP-dependent RNA helicase DExH12 |
| 141 | A0A218WVG2 | Uncharacterized protein LOC116201738 | 633823.67 | 116528.5 | 488586.67 | 41.25 | 2442780 | 226.99 | 2.53×10^-8^ | uncharacterized protein LOC116201738 |
| 142 | A0A6P8BT43 | Eukaryotic peptide chain release factor GTP-binding subunit ERF3A-like isoform X1 | 260798 | 41.25 | 289559.67 | 41.25 | 479417.33 | 225.65 | 2.59×10^-8^ | eukaryotic peptide chain release factor GTP-binding subunit ERF3A-like isoform X1 |
| 143 | A0A218X4I2 | SGS domain-containing protein | 151821.67 | 41.25 | 131931.33 | 41.25 | 96888.63 | 224.37 | 2.64×10^-8^ | hypothetical protein CDL15-Pgr023008 |
| 144 | A0A218XAE1 | K Homology domain-containing protein | 1291063.33 | 41.25 | 1402316.67 | 41.25 | 817094.67 | 223.75 | 2.64×10^-8^ | hypothetical protein CDL15-Pgr007943 |
| 145 | A0A218XUM4 | Proton pump-interactor 1-like | 1681366.67 | 41.25 | 2195400 | 41.25 | 1317490 | 223.9 | 2.64×10^-8^ | proton pump-interactor 1-like |
| 146 | A0A218WA16 | V-type proton ATPase catalytic subunit A | 167087.33 | 200241 | 351472.33 | 279162.33 | 1125303.33 | 222.74 | 2.66×10^-8^ | V-type proton ATPase catalytic subunit A |
| 147 | A0A2I0IVR0 | Huntingtin-interacting protein K | 637379 | 41.25 | 576125.33 | 41.25 | 286834.33 | 222.86 | 2.66×10^-8^ | huntingtin-interacting protein K |
| 148 | A0A218W785 | Aspartyl protease family protein 2 | 41.25 | 41.25 | 41.25 | 543614.67 | 41.25 | 221.86 | 2.70×10^-8^ | aspartyl protease family protein 2 |
| 149 | A0A218X1Q3 | Transcription elongation factor spt6 | 41.25 | 41.25 | 41.25 | 41.25 | 47475.87 | 220.48 | 2.76×10^-8^ | hypothetical protein CDL15-Pgr003044 |
| 150 | A0A2I0J9N3 | Poly [ADP-ribose] polymerase tankyras× 101 | 88667.03 | 41.25 | 41.25 | 41.25 | 436299.33 | 219.75 | 2.79×10^-8^ | poly [ADP-ribose] polymerase tankyrase-1 |
| 151 | A0A218WF09 | 20 kDa chaperonin, chloroplastic | 912511.33 | 41.25 | 573159 | 41.25 | 559729 | 219.38 | 2.79×10^-8^ | hypothetical protein CDL15-Pgr011195 |
| 152 | A0A218W7U8 | V-type proton ATPase subunit a | 7523.3 | 41.25 | 41.25 | 41.25 | 9372.78 | 217.2 | 2.91×10^-8^ | hypothetical protein CDL15-Pgr025052 |
| 153 | A0A218WPT9 | Protein TIME FOR COFFEE | 96953.9 | 41.25 | 41.25 | 41.25 | 41.25 | 216.7 | 2.91×10^-8^ | hypothetical protein CDL15-Pgr008555 |
| 154 | A0A218X325 | Protein TIC 21, chloroplastic | 8587.3 | 41.25 | 41.25 | 41.25 | 497047.67 | 216.92 | 2.91×10^-8^ | protein TIC 21, chloroplastic |
| 155 | A0A218WAN6 | Late embryogenesis abundant protein B19.1A | 261421000 | 12478500 | 302284000 | 4813096.67 | 37736733.33 | 214 | 3.08×10^-8^ | late embryogenesis abundant protein B19.1A |
| 156 | A0A218W322 | Protein RETICULATA-RELATED 3, chloroplastic-like | 877813.67 | 41.25 | 2140073.33 | 41.25 | 241857.33 | 209.72 | 3.38×10^-8^ | protein RETICULATA-RELATED 3, chloroplastic-like |
| 157 | A0A2I0IJP7 | V-type proton ATPase subunit c''2 | 3042.81 | 41.25 | 41.25 | 41.25 | 45429.5 | 209.31 | 3.39×10^-8^ | V-type proton ATPase subunit c''2 |
| 158 | A0A218XTG2 | Light-harvesting complex-like protein 3 isotype 2, chloroplastic | 66743.73 | 41.25 | 41.25 | 41.25 | 158181.67 | 208.94 | 3.40×10^-8^ | light-harvesting complex-like protein 3 isotype 2, chloroplastic |
| 159 | A0A218W461 | RBR-type E3 ubiquitin transferase | 41.25 | 41.25 | 41.25 | 41.25 | 38273.67 | 208.08 | 3.44×10^-8^ | probable E3 ubiquitin-protein ligase ARI7 isoform X2 |
| 160 | A0A6P8DVC6 | RNA helicase | 680528.33 | 41.25 | 506075.67 | 228439.67 | 112508.63 | 207.87 | 3.44×10^-8^ | DEAD-box ATP-dependent RNA helicase 38 |
| 161 | A0A218W2A1 | Cinnamoyl-CoA reductase 1-like | 1392140 | 511944.33 | 1039203.33 | 730159 | 2938920 | 206.11 | 3.56×10^-8^ | cinnamoyl-CoA reductase 1-like |
| 162 | A0A218XZU4 | Aminoacylas× 101 | 20887.9 | 41.25 | 41.25 | 41.25 | 12071.47 | 205.55 | 3.59×10^-8^ | aminoacylase-1 |
| 163 | A0A218WYP0 | SMP domain-containing protein | 2921886.67 | 583253 | 1267670 | 41.25 | 915081.67 | 204.29 | 3.68×10^-8^ | hypothetical protein CDL15-Pgr018411 |
| 164 | A0A218X6Q7 | 3-phosphoshikimate 1-carboxyvinyltransferase | 184790.33 | 41.25 | 41.25 | 41.25 | 102301.7 | 203.6 | 3.72×10^-8^ | hypothetical protein CDL15-Pgr019355 |
| 165 | A0A218WXI2 | Protein MOR1 | 41.25 | 41.25 | 41.25 | 41.25 | 13121.2 | 203.02 | 3.75×10^-8^ | hypothetical protein CDL15-Pgr016894 |
| 166 | A0A218WAN3 | alpha-1,2-Mannosidase | 41.25 | 41.25 | 41.25 | 41.25 | 28220.3 | 202.33 | 3.79×10^-8^ | hypothetical protein CDL15-Pgr013969 |
| 167 | A0A218VXJ2 | Vacuolar protein sorting-associated protein 32 homolog 2-like | 108584 | 41.25 | 41.25 | 41.25 | 101577.9 | 201.85 | 3.81×10^-8^ | vacuolar protein sorting-associated protein 32 homolog 2-like |
| 168 | A0A218WG86 | Proteasome subunit beta | 56978.4 | 41.25 | 41.25 | 41.25 | 62592.43 | 200.6 | 3.90×10^-8^ | proteasome subunit beta type-7-A-like |
| 169 | A0A218W571 | ENTH domain-containing protein | 92028.33 | 41.25 | 116839 | 41.25 | 75399 | 200.22 | 3.92×10^-8^ | hypothetical protein CDL15-Pgr024754 |
| 170 | A0A218Y2I1 | Probable cinnamyl alcohol dehydrogenase 9 | 24582.7 | 41.25 | 49182.67 | 74314.4 | 25071.93 | 198.89 | 4.02×10^-8^ | probable cinnamyl alcohol dehydrogenase 9 |
| 171 | A0A218WGD1 | CASP-like protein | 8056.95 | 41.25 | 41.25 | 41.25 | 41.25 | 198.33 | 4.06×10^-8^ | CASP-like protein 4A3 |
| 172 | A0A218WTP6 | Protein CURVATURE THYLAKOID 1A, chloroplastic-like | 605.91 | 41.25 | 41.25 | 41.25 | 15344.53 | 198.09 | 4.06×10^-8^ | protein CURVATURE THYLAKOID 1A, chloroplastic-like |
| 173 | A0A218W821 | Uncharacterized protein LOC116192991 | 36848.1 | 41.25 | 41.25 | 41.25 | 27428.7 | 196.58 | 4.16×10^-8^ | uncharacterized protein LOC116192991 |
| 174 | A0A218WQ98 | NAD(P)H dehydrogenase (quinone) | 23042.57 | 41.25 | 41.25 | 41.25 | 43364.03 | 196.66 | 4.16×10^-8^ | hypothetical protein CDL15-Pgr00510-9 |
| 175 | A0A218W8T2 | Uncharacterized protein LOC116211817 | 41.25 | 41.25 | 41.25 | 41.25 | 64679.5 | 194.54 | 4.33×10^-8^ | uncharacterized protein LOC116211817 |
| 176 | A0A218XYY4 | Signal recognition particle subunit SRP72 | 19287.63 | 41.25 | 41.25 | 41.25 | 61426.13 | 194.71 | 4.33×10^-8^ | hypothetical protein CDL15-Pgr024479 |
| 177 | A0A218WS67 | Cell number regulator 6-like | 41.25 | 41.25 | 41.25 | 41.25 | 51067.4 | 193.23 | 4.44×10^-8^ | hypothetical protein CDL15-Pgr021862 |
| 178 | A0A218X316 | phosphoribosylamin× 10-glycine ligase | 122530.67 | 41.25 | 41.25 | 41.25 | 203526.33 | 193.13 | 4.44×10^-8^ | phosphoribosylamine--glycine ligase |
| 179 | A0A218WMJ7 | YTH domain-containing family protein | 32584.83 | 41.25 | 41.25 | 41.25 | 59132.6 | 190.98 | 4.59×10^-8^ | hypothetical protein CDL15-Pgr001346 |
| 180 | A0A218X6B2 | Ubiquitin domain-containing protein DSK2b-like isoform X2 | 173117 | 41.25 | 147161.33 | 41.25 | 396799.67 | 191.18 | 4.59×10^-8^ | ubiquitin domain-containing protein DSK2b-like isoform X2 |
| 181 | A0A218XIY6 | Metalloenzyme domain-containing protein | 39919 | 41.25 | 41.25 | 41.25 | 35177.57 | 191.52 | 4.59×10^-8^ | hypothetical protein CDL15-Pgr027473 |
| 182 | A0A2I0JHK7 | Protein seele | 198886.33 | 41.25 | 207456.67 | 41.25 | 499550.67 | 191.06 | 4.59×10^-8^ | protein seele |
| 183 | A0A218VQD1 | Uncharacterized protein | 39054.33 | 41.25 | 30977.97 | 41.25 | 226276 | 190.64 | 4.60×10^-8^ | hypothetical protein CDL15-Pgr020031 |
| 184 | A0A218WL36 | Peroxisomal and mitochondrial division factor 2-like | 687483.67 | 359206 | 609263.33 | 471826.33 | 2523696.67 | 190.44 | 4.60×10^-8^ | peroxisomal and mitochondrial division factor 2-like |
| 185 | A0A6P8BMR5 | dCTP pyrophosphatase 1 | 56108.13 | 41.25 | 41.25 | 41.25 | 88091.83 | 190.06 | 4.63×10^-8^ | dCTP pyrophosphatase 1 |
| 186 | A0A218WXL0 | Transmembrane 9 superfamily member | 67.75 | 41.25 | 41.25 | 41.25 | 487.87 | 189.64 | 4.65×10^-8^ | transmembrane 9 superfamily member 7 |
| 187 | A0A218WZV1 | Cytochrome c oxidase subunit 5b-1, mitochondrial-like | 95062.43 | 41556.17 | 234816.33 | 41.25 | 1224480 | 187.69 | 4.87×10^-8^ | hypothetical protein CDL15-Pgr015063 |
| 188 | A0A218XFG3 | SKP1-like protein | 276923 | 24512.5 | 41.25 | 41.25 | 737404 | 185.89 | 5.08×10^-8^ | SKP1-like protein 1 |
| 189 | A0A218X0T7 | Uncharacterized protein LOC116194096 | 19176.23 | 41.25 | 41.25 | 41.25 | 378549.33 | 184.59 | 5.23×10^-8^ | uncharacterized protein LOC116194096 |
| 190 | A0A218XXF8 | RuBisCO large subunit-binding protein subunit beta, chloroplastic | 41.25 | 41.25 | 41.25 | 41.25 | 6280.53 | 183.01 | 5.40×10^-8^ | hypothetical protein CDL15-Pgr024594 |
| 191 | A0A6P8BPS0 | Alanin× 10-tRNA ligase | 124917.33 | 41.25 | 159518 | 41.25 | 137031.67 | 182.99 | 5.40×10^-8^ | alanine--tRNA ligase |
| 192 | A0A218XJ54 | Glycin× 10rich RNA-binding protein RZ1A | 26491.17 | 41.25 | 41.25 | 41.25 | 82096.03 | 182.37 | 5.46×10^-8^ | hypothetical protein CDL15-Pgr027731 |
| 193 | A0A218VXN4 | Complex 1 LYR protein domain-containing protein | 75410.2 | 41.25 | 5277.27 | 41.25 | 139710.67 | 181.06 | 5.63×10^-8^ | hypothetical protein CDL15-Pgr008835 |
| 194 | A0A218VWB9 | Pr× 10mRNA-processing protein 40A | 132467.67 | 41.25 | 41.25 | 41.25 | 95072.63 | 179.19 | 5.89×10^-8^ | hypothetical protein CDL15-Pgr028492 |
| 195 | A0A2I0IM77 | Ketoreductase domain-containing protein | 41.25 | 41.25 | 41.25 | 41.25 | 343702.33 | 178.69 | 5.94×10^-8^ | hypothetical protein CRG98-034486 |
| 196 | A0A2I0JMT8 | Vicilin-like seed storage protein At2g18540 | 940283 | 83507.75 | 1240986.67 | 41.25 | 389295.33 | 177.95 | 6.04×10^-8^ | vicilin-like seed storage protein At2g18540 |
| 197 | A0A2I0KJC9 | Methyl-CpG-binding domain-containing protein 4 | 46554.4 | 41.25 | 41.25 | 41.25 | 81709.2 | 175.47 | 6.40×10^-8^ | methyl-CpG-binding domain-containing protein 4 |
| 198 | A0A2I0KU37 | Uncharacterized protein LOC116199663 | 113678.67 | 41.25 | 103549.43 | 41.25 | 116366.67 | 175.63 | 6.40×10^-8^ | uncharacterized protein LOC116199663 |
| 199 | A0A6P8BRJ7 | Nudix hydrolase 26, chloroplastic | 447137.67 | 807714.67 | 79732.75 | 41.25 | 161110 | 175.24 | 6.41×10^-8^ | nudix hydrolase 26, chloroplastic |
| 200 | A0A218XCU3 | D-lactate dehydrogenase (cytochrome) | 41.25 | 41.25 | 41.25 | 41.25 | 41056.07 | 173.96 | 6.58×10^-8^ | hypothetical protein CDL15-Pgr002193 |
| 201 | A0A218XHX3 | Villin-4-like | 119324.67 | 139130.33 | 124899.33 | 41.25 | 239184.33 | 173.96 | 6.58×10^-8^ | villin-4-like |
| 202 | A0A218WU80 | Probable 6-phosphogluconolactonase | 2659106.67 | 482751.33 | 425033.33 | 832674.33 | 986334 | 173.29 | 6.67×10^-8^ | probable 6-phosphogluconolactonase 4, chloroplastic |
| 203 | A0A218WDL1 | NADP-dependent oxidoreductase domain-containing protein | 4670856.67 | 583999.67 | 753357.67 | 1896006.67 | 1822066.67 | 172.61 | 6.77×10^-8^ | hypothetical protein CDL15-Pgr013145 |
| 204 | A0A218WJ02 | Importin subunit alpha | 9725.08 | 41.25 | 41.25 | 41.25 | 23582.87 | 171.81 | 6.88×10^-8^ | importin subunit alpha-1-like |
| 205 | A0A218WZK7 | glutathione transferase | 8195776.67 | 1253186.67 | 1470230 | 1603993.33 | 2600476.67 | 171.69 | 6.88×10^-8^ | glutathione S-transferase-like |
| 206 | A0A218W7X8 | 60S ribosomal protein L12 | 1127263.33 | 350729 | 4917660 | 1518456.67 | 1554643.33 | 171.32 | 6.92×10^-8^ | hypothetical protein CDL15-Pgr025082 |
| 207 | A0A218XTW3 | Inositol-tetrakisphosphate 1-kinase | 16144.5 | 41.25 | 41.25 | 41.25 | 8417.33 | 170.42 | 7.07×10^-8^ | inositol-tetrakisphosphate 1-kinase 1 |
| 208 | A0A2I0LDP6 | Cyclin-dependent kinases regulatory subunit | 5689.27 | 41.25 | 41.25 | 41.25 | 15500.93 | 169.16 | 7.30×10^-8^ | cyclin-dependent kinases regulatory subunit 1 |
| 209 | A0A2I0HV12 | Major latex allergen Hev b 5-like | 766756.33 | 218487.67 | 631270.33 | 41.25 | 42226.9 | 168.43 | 7.42×10^-8^ | hypothetical protein CRG98-043978 |
| 210 | A0A218WZE7 | Phospholipase D | 28917 | 44959.67 | 20781.15 | 363835 | 34973.33 | 167.48 | 7.59×10^-8^ | phospholipase D alpha 1 |
| 211 | A0A6P8CCH2 | Elongation factor 1-delta 1-like | 8220360 | 1854178.67 | 3806043.33 | 908790.83 | 19662333.33 | 166.06 | 7.88×10^-8^ | elongation factor 1-delta 1-like |
| 212 | A0A218X0D1 | Essential protein Yae1 N-terminal domain-containing protein | 7137.9 | 41.25 | 41.25 | 41.25 | 14308.3 | 165.62 | 7.94×10^-8^ | hypothetical protein CDL15-Pgr016158 |
| 213 | A0A218XQZ8 | Laccase | 3414.44 | 1059.32 | 4995.01 | 26131.5 | 2150.3 | 164.87 | 8.08×10^-8^ | laccase-12-like isoform X2 |
| 214 | A0A218W4I1 | GOLD domain-containing protein | 21520.53 | 41.25 | 41.25 | 41.25 | 31198.77 | 164.63 | 8.10×10^-8^ | hypothetical protein CDL15-Pgr000464 |
| 215 | A0A218VS70 | Acyl-CoA-binding domain-containing protein 4-like | 128533.33 | 41.25 | 82155.53 | 41.25 | 156192 | 163.7 | 8.29×10^-8^ | acyl-CoA-binding domain-containing protein 4-like |
| 216 | A0A218W759 | Pectinesterase | 65820.23 | 41.25 | 41.25 | 41.25 | 132350.33 | 163.22 | 8.31×10^-8^ | pectinesterase-like |
| 217 | A0A218X709 | Protein ALTERED PHOSPHATE STARVATION RESPONSE 1-like isoform X2 | 41.25 | 41.25 | 41.25 | 41.25 | 7276.79 | 163.29 | 8.31×10^-8^ | protein ALTERED PHOSPHATE STARVATION RESPONSE 1-like isoform X2 |
| 218 | A0A2I0HSK3 | Uncharacterized protein | 38104.97 | 41.25 | 41.25 | 41.25 | 10415.43 | 163.18 | 8.31×10^-8^ | hypothetical protein CRG98-045130 |
| 219 | A0A218XZW3 | Protein NUCLEAR FUSION DEFECTIVE 2 | 1058233.33 | 4931043.33 | 1006399.33 | 789176.67 | 975745.33 | 162.88 | 8.34×10^-8^ | protein NUCLEAR FUSION DEFECTIVE 2 |
| 220 | A0A2I0J3A9 | Tripeptidyl peptidase II Ig-like domain-containing protein (Fragment) | 413308.67 | 140003.42 | 553618.67 | 41.25 | 2185273.33 | 162.76 | 8.34×10^-8^ | hypothetical protein CRG98-028861, partial |
| 221 | A0A2I0K371 | Uncharacterized protein LOC116201789 | 16548900 | 40188033.33 | 12111100 | 134516.5 | 961123.67 | 162.59 | 8.34×10^-8^ | uncharacterized protein LOC116201789 |
| 222 | A0A218VXE9 | SNAP25 homologous protein SNAP33-like | 50039.43 | 41.25 | 41.25 | 41.25 | 82687.47 | 161.9 | 8.48×10^-8^ | SNAP25 homologous protein SNAP33-like |
| 223 | A0A2I0K2C8 | Annexin | 1361876.67 | 912512.33 | 907394.33 | 2374803.33 | 5383803.33 | 160.47 | 8.78×10^-8^ | hypothetical protein CRG98-016892 |
| 224 | A0A2I0L329 | Altered inheritance of mitochondria protein 32-like | 47249.07 | 41.25 | 41.25 | 41.25 | 90622.33 | 160.57 | 8.78×10^-8^ | altered inheritance of mitochondria protein 32-like |
| 225 | A0A218WED1 | Monosaccharid× 10sensing protein 2-like | 50299.33 | 41.25 | 41.25 | 41.25 | 188693.33 | 158.87 | 9.16×10^-8^ | monosaccharide-sensing protein 2-like |
| 226 | A0A6P8D9P4 | adenylate kinase | 47504.67 | 41.25 | 41.25 | 41.25 | 45797.83 | 158.79 | 9.16×10^-8^ | UMP-CMP kinase isoform X1 |
| 227 | A0A218WMZ3 | Uncharacterized protein | 77777.97 | 41.25 | 41.25 | 41.25 | 51654.17 | 157.28 | 9.56×10^-8^ | hypothetical protein CDL15-Pgr001486 |
| 228 | A0A218W3U7 | 11-beta-hydroxysteroid dehydrogenas× 10like 5 | 170529.33 | 1133593.33 | 821259.33 | 3578190 | 914711.33 | 156.76 | 9.58×10^-8^ | 11-beta-hydroxysteroid dehydrogenase-like 5 |
| 229 | A0A218WKQ5 | Protein TIC 40, chloroplastic | 735380.67 | 41.25 | 720629.67 | 41.25 | 624879.33 | 156.67 | 9.58×10^-8^ | hypothetical protein CDL15-Pgr001340 |
| 230 | A0A2I0JF86 | GDSL esterase/lipase At2g30310-like isoform X1 | 2945803.33 | 11520000 | 3472013.33 | 2444890 | 1775943.33 | 156.86 | 9.58×10^-8^ | GDSL esterase/lipase At2g30310-like isoform X1 |
| 231 | A0A2I0KK40 | Pyruvate, phosphate dikinase | 2025876.67 | 1080056.67 | 1230850 | 1762853.33 | 3400803.33 | 157.07 | 9.58×10^-8^ | hypothetical protein CRG98-010866 |
| 232 | A0A218XUJ0 | Late embryogenesis abundant protein 18 | 2382800 | 327602.75 | 4911733.33 | 151469.17 | 2674113.33 | 156.43 | 9.61×10^-8^ | late embryogenesis abundant protein 18 |
| 233 | A0A218XKK6 | UDP-glucose 4-epimerase | 237985.33 | 41.25 | 190727 | 41.25 | 250066 | 154.99 | 1.00×10^-7^ | hypothetical protein CDL15-Pgr028006 |
| 234 | A0A218XGH3 | Actin cytoskeleton-regulatory complex protein PAN1-like | 82325.17 | 41.25 | 102238.67 | 41.25 | 106125.33 | 154.65 | 1.01×10^-7^ | actin cytoskeleton-regulatory complex protein PAN1-like |
| 235 | A0A218X9M2 | Non-specific lipid-transfer protein | 25268300 | 135339000 | 55630000 | 1101197.08 | 1696413.33 | 153.66 | 1.04×10^-7^ | hypothetical protein CDL15-Pgr007671 |
| 236 | A0A218VTD9 | RRM domain-containing protein | 29173.32 | 865818 | 252476.33 | 127490.33 | 101008.27 | 153.4 | 1.04×10^-7^ | hypothetical protein CDL15-Pgr019499 |
| 237 | A0A218X4J5 | Factor of DNA methylation 1-like isoform X1 | 504986 | 322739.67 | 577730.33 | 232695.75 | 2155703.33 | 153.09 | 1.05×10^-7^ | factor of DNA methylation 1-like isoform X1 |
| 238 | A0A2I0KJG4 | PSME3-interacting protein | 22992.27 | 41.25 | 41.25 | 41.25 | 31598.13 | 152.72 | 1.05×10^-7^ | PSME3-interacting protein |
| 239 | A0A2I0IEF8 | 5'-deoxynucleotidase | 252805.33 | 41.25 | 11659.93 | 41.25 | 137283 | 152.51 | 1.06×10^-7^ | hypothetical protein CRG98-037249 |
| 240 | A0A218VZ99 | beta-aspartyl-peptidase | 218788.67 | 207582.33 | 225617 | 41.25 | 201256.67 | 152.04 | 1.06×10^-7^ | isoaspartyl peptidase/L-asparaginase |
| 241 | A0A218X298 | Stromal 70 kDa heat shock-related protein, chloroplastic | 896717.67 | 174195.5 | 548676.67 | 41.25 | 1237890 | 152.02 | 1.06×10^-7^ | stromal 70 kDa heat shock-related protein, chloroplastic |
| 242 | A0A218WIU0 | PUA domain-containing protein | 396.82 | 41.25 | 41.25 | 645.1 | 897.21 | 151.16 | 1.09×10^-7^ | hypothetical protein CDL15-Pgr018348 |
| 243 | A0A218XGR1 | Peptidyl-prolyl cis-trans isomerase | 8830653.33 | 2245550 | 2781843.33 | 4443826.67 | 7430313.33 | 150.57 | 1.11×10^-7^ | peptidyl-prolyl cis-trans isomerase |
| 244 | A0A218XE76 | 60S ribosomal protein L21-1-like | 41.25 | 41.25 | 41.25 | 19940.47 | 600.04 | 150.03 | 1.12×10^-7^ | 60S ribosomal protein L21-1-like |
| 245 | A0A218Y1Q2 | DNA-directed RNA polymerase subunit beta | 41.25 | 41.25 | 41.25 | 4307.99 | 41.25 | 150.01 | 1.12×10^-7^ | DNA-directed RNA polymerase II subunit RPB2 |
| 246 | A0A2I0HZ29 | 60S ribosomal protein L37a | 6009.44 | 41.25 | 44203.3 | 41.25 | 10166.46 | 149.6 | 1.12×10^-7^ | hypothetical protein CRG98-042675 |
| 247 | A0A2I0KBM9 | GYF domain-containing protein | 1638376.67 | 41.25 | 1924523.33 | 41.25 | 829548.67 | 149.7 | 1.12×10^-7^ | hypothetical protein CRG98-013661 |
| 248 | A0A2I0K5S5 | Probable NADH dehydrogenase [ubiquinone] 1 alpha subcomplex subunit 5, mitochondrial | 638538.67 | 651584 | 388861.67 | 185290.83 | 3386463.33 | 147.69 | 1.19×10^-7^ | probable NADH dehydrogenase [ubiquinone] 1 alpha subcomplex subunit 5, mitochondrial |
| 249 | A0A218WQW7 | 20 kDa chaperonin, chloroplastic | 4366266.67 | 1507746.67 | 1531483.33 | 193553.08 | 3213520 | 147.08 | 1.20×10^-7^ | 20 kDa chaperonin, chloroplastic-like |
| 250 | A0A218X1Z8 | CDK5RAP3-like protein | 25559.13 | 41.25 | 8999.87 | 41.25 | 152379.67 | 147.1 | 1.20×10^-7^ | CDK5RAP3-like protein |
| 251 | A0A218XVK7 | glutathione transferase | 185309.33 | 41.25 | 41.25 | 41.25 | 98697 | 147.13 | 1.20×10^-7^ | probable glutathione S-transferase parA |
| 252 | A0A6P8E503 | Probable glucan 1,3-alpha-glucosidase | 41.25 | 41.25 | 41.25 | 41.25 | 112707.33 | 147.44 | 1.20×10^-7^ | probable glucan 1,3-alpha-glucosidase isoform X1 |
| 253 | A0A6P8DMN0 | Tripeptidyl-peptidase 2 | 1403763.33 | 1312386.67 | 2448946.67 | 4162683.33 | 4720323.33 | 146.95 | 1.20×10^-7^ | tripeptidyl-peptidase 2 |
| 254 | A0A2I0HSN4 | Far upstream element-binding protein 2 | 2007463.33 | 422631.67 | 2113720 | 41.25 | 1311563.33 | 146.77 | 1.20×10^-7^ | far upstream element-binding protein 2 |
| 255 | A0A218X3C4 | Universal stress protein A-like protein isoform X2 | 5573686.67 | 2099850 | 1727383.33 | 2119130 | 5344436.67 | 146.22 | 1.21×10^-7^ | universal stress protein A-like protein isoform X2 |
| 256 | A0A218XJC7 | E2 ubiquitin-conjugating enzyme | 14191.63 | 41.25 | 41.25 | 41.25 | 42747.1 | 146.2 | 1.21×10^-7^ | hypothetical protein CDL15-Pgr028092 |
| 257 | A0A6P8DSV9 | Late embryogenesis abundant protein D-29-like isoform X1 | 8750670 | 1374416.67 | 6959776.67 | 41.25 | 10-914866.67 | 145.57 | 1.23×10^-7^ | late embryogenesis abundant protein D-29-like isoform X2 |
| 258 | A0A218VU82 | non-specific serine/threonine protein kinase | 41.25 | 41.25 | 41.25 | 41.25 | 9554.11 | 145.09 | 1.24×10^-7^ | hypothetical protein CDL15-Pgr008124 |
| 259 | A0A218X898 | EF-hand domain-containing protein | 75860.93 | 12583.37 | 462302 | 41.25 | 106320 | 145.13 | 1.24×10^-7^ | hypothetical protein CDL15-Pgr007473 |
| 260 | A0A2I0KJQ7 | Nascent polypeptid× 10associated complex subunit alpha-like protein 1 | 5283866.67 | 1418143.33 | 3195650 | 1127283 | 897707.33 | 145.01 | 1.24×10^-7^ | nascent polypeptide-associated complex subunit alpha-like protein 1 |
| 261 | A0A218WAP2 | Uncharacterized protein LOC116195164 | 41.25 | 41.25 | 41.25 | 41.25 | 1127.39 | 142.66 | 1.34×10^-7^ | uncharacterized protein LOC116195164 |
| 262 | A0A218XHW0 | inositol-1,3,4-trisphosphate 5/6-kinase | 62248.53 | 41.25 | 41.25 | 41.25 | 39831.47 | 142.62 | 1.34×10^-7^ | inositol 1,3,4-trisphosphate 5/6-kinase 4 isoform X2 |
| 263 | A0A218WXM1 | Late embryogenesis abundant protein At3g53040-like isoform X2 | 52768400 | 4038300 | 20760966.67 | 1607660.42 | 36981800 | 141.87 | 1.37×10^-7^ | late embryogenesis abundant protein At3g53040-like isoform X2 |
| 264 | A0A2I0L1Q5 | S12 | 5088.63 | 2256.99 | 4103.89 | 95122.6 | 3888.52 | 141.21 | 1.40×10^-7^ | hypothetical protein CRG98-004958 |
| 265 | A0A2I0JJW8 | T-complex protein 1 subunit gamma | 168730 | 41.25 | 151056.33 | 228154 | 241022.33 | 141.03 | 1.40×10^-7^ | hypothetical protein CRG98-023085 |
| 266 | A0A218X683 | transketolase | 59556.33 | 41.25 | 96795.27 | 41.25 | 94386.57 | 140.43 | 1.42×10^-7^ | transketolase, chloroplastic |
| 267 | A0A218WHC7 | Expansin | 41.25 | 41.25 | 41.25 | 465463.67 | 1156.62 | 139.56 | 1.46×10^-7^ | hypothetical protein CDL15-Pgr017846 |
| 268 | A0A218WG59 | FAM10 family protein At4g22670 | 5570070 | 842777.33 | 1699293.33 | 210-996.75 | 7878626.67 | 137.95 | 1.54×10^-7^ | FAM10 family protein At4g22670 |
| 269 | A0A2I0IZ97 | Uncharacterized protein LOC116198135 | 41.25 | 41.25 | 41.25 | 41.25 | 9899.28 | 137.91 | 1.54×10^-7^ | uncharacterized protein LOC116198135 |
| 270 | A0A218XA97 | Vacuolar-sorting receptor 1-like | 519344.67 | 663688.67 | 354004.67 | 307959 | 842525 | 137.51 | 1.55×10^-7^ | vacuolar-sorting receptor 1-like |
| 271 | A0A2I0JMH5 | alcohol dehydrogenase | 2234253.33 | 578461.67 | 1089970.33 | 916421 | 2672983.33 | 136 | 1.63×10^-7^ | alcohol dehydrogenase 1 |
| 272 | A0A218X9C1 | 14-3-3-like protein | 3026690 | 614210 | 665811.67 | 648191 | 3926190 | 135.34 | 1.67×10^-7^ | 14-3-3-like protein |
| 273 | A0A2I0L1Z4 | Ureidoglycolate hydrolase | 58359.83 | 41.25 | 41.25 | 41.25 | 33174.27 | 135.03 | 1.67×10^-7^ | hypothetical protein CRG98-004957 |
| 274 | A0A2I0L603 | UspA domain-containing protein | 12353233.33 | 3098226.67 | 14992300 | 1750753.33 | 21750100 | 135.05 | 1.67×10^-7^ | hypothetical protein CRG98-003510 |
| 275 | A0A218WB34 | DOG1 domain-containing protein | 17423.03 | 41.25 | 41.25 | 41.25 | 43571.07 | 134.75 | 1.68×10^-7^ | hypothetical protein CDL15-Pgr013872 |
| 276 | A0A218X9B9 | Lipoyl-binding domain-containing protein | 46186.3 | 41.25 | 41.25 | 41.25 | 31617.6 | 134.77 | 1.68×10^-7^ | hypothetical protein CDL15-Pgr007863 |
| 277 | A0A218WX77 | GB1/RHD3-type G domain-containing protein | 41.25 | 41.25 | 41.25 | 41.25 | 36412.83 | 134.11 | 1.71×10^-7^ | hypothetical protein CDL15-Pgr013210 |
| 278 | A0A218VY06 | LIM zinc-binding domain-containing protein | 95047.93 | 88255.75 | 153405 | 297719.67 | 1002168.67 | 133.87 | 1.72×10^-7^ | hypothetical protein CDL15-Pgr008965 |
| 279 | A0A218WDU4 | THO complex subunit 7B-like | 57729.2 | 41.25 | 41.25 | 41.25 | 237573.33 | 133.83 | 1.72×10^-7^ | THO complex subunit 7B-like |
| 280 | A0A218VVR3 | Abscisic stress-ripening protein 1-like | 853481 | 1553861.67 | 1302180 | 227276.42 | 7627740 | 133.45 | 1.73×10^-7^ | abscisic stress-ripening protein 1-like |
| 281 | A0A218VW67 | Cysteine synthase | 3249.89 | 41.25 | 41.25 | 41.25 | 52379.07 | 132.06 | 1.81×10^-7^ | bifunctional L-3-cyanoalanine synthase/cysteine synthase 2, mitochondrial |
| 282 | A0A218X829 | Metacaspas× 104 | 672419 | 317760.33 | 611421.67 | 41.25 | 1258056.67 | 132.13 | 1.81×10^-7^ | metacaspase-4 |
| 283 | A0A218VRW1 | peptidylprolyl isomerase | 3234493.33 | 570039 | 4293323.33 | 121724.5 | 1910330 | 131.88 | 1.82×10^-7^ | FK506-binding protein 2-like |
| 284 | A0A6P8D4L2 | Calreticulin | 1142666.67 | 717207.33 | 445907 | 580156.33 | 1753843.33 | 131.18 | 1.86×10^-7^ | calreticulin |
| 285 | A0A218W0K9 | Acidic leucin× 10rich nuclear phosphoprotein 32-related protein | 35681.7 | 41.25 | 41.25 | 41.25 | 31825 | 130.82 | 1.88×10^-7^ | acidic leucine-rich nuclear phosphoprotein 32-related protein |
| 286 | A0A6P8C7A2 | Uncharacterized protein LOC116193021 isoform X2 | 1801073.33 | 507844.67 | 1299396.67 | 54556.17 | 2297966.67 | 130.38 | 1.90×10^-7^ | uncharacterized protein LOC116193021 isoform X2 |
| 287 | A0A218X3M2 | Low-temperatur× 10induced 65 kDa protein-like isoform X1 | 6889586.67 | 749452.33 | 7702766.67 | 533491 | 4892486.67 | 129.75 | 1.94×10^-7^ | low-temperature-induced 65 kDa protein-like isoform X1 |
| 288 | A0A218XN76 | Cryptochrom× 101 | 41.25 | 41.25 | 41.25 | 41.25 | 46784.4 | 129.77 | 1.94×10^-7^ | cryptochrome-1 |
| 289 | A0A218VVD7 | Riboflavin biosynthesis protein PYRD, chloroplastic | 24125.5 | 41.25 | 41.25 | 41.25 | 40135.83 | 129.16 | 1.97×10^-7^ | hypothetical protein CDL15-Pgr018811 |
| 290 | A0A218VR53 | Uncharacterized protein LOC116212689 | 436044.33 | 41.25 | 156800 | 41.25 | 172600 | 128.45 | 2.00×10^-7^ | uncharacterized protein LOC116212689 |
| 291 | A0A218WAK9 | Late embryogenesis abundant protein D-34-like | 14980733.33 | 2438430 | 6187103.33 | 612167.5 | 1195266.67 | 128.58 | 2.00×10^-7^ | late embryogenesis abundant protein D-34-like |
| 292 | A0A218X3M8 | PHD finger protein ALFIN-LIKE | 33423.37 | 41.25 | 41.25 | 41.25 | 42523.17 | 128.49 | 2.00×10^-7^ | hypothetical protein CDL15-Pgr003469 |
| 293 | A0A218X5A1 | 14-3-3-like protein | 1034092 | 90799.95 | 333215.67 | 41.25 | 1311643.33 | 127.94 | 2.04×10^-7^ | 14-3-3-like protein |
| 294 | A0A218VV27 | TOM1-like protein 3 | 1683.52 | 41.25 | 41.25 | 41.25 | 3478.01 | 127.75 | 2.04×10^-7^ | TOM1-like protein 3 |
| 295 | A0A218WDU2 | Peroxiredoxin | 28449933.33 | 3685903.33 | 4654733.33 | 1503704.33 | 32742166.67 | 127.81 | 2.04×10^-7^ | 1-Cys peroxiredoxin |
| 296 | A0A218WB18 | Uncharacterized protein | 1847663.33 | 544830.33 | 745732.33 | 41.25 | 1588393.33 | 126.24 | 2.15×10^-7^ | hypothetical protein CDL15-Pgr025497 |
| 297 | A0A218XNN5 | Beta-galactosidase | 91693.93 | 372690 | 10-9163.27 | 41.25 | 54393.77 | 126.33 | 2.15×10^-7^ | hypothetical protein CDL15-Pgr026416 |
| 298 | A0A218W933 | Poly [ADP-ribose] polymerase | 1266783.33 | 670250 | 1114230 | 786538 | 1796523.33 | 126.03 | 2.15×10^-7^ | hypothetical protein CDL15-Pgr025186 |
| 299 | A0A2I0K0T1 | glutathione transferase | 2275496.67 | 531453.67 | 365219.33 | 895660.33 | 614148.33 | 125.95 | 2.15×10^-7^ | hypothetical protein CRG98-017547 |
| 300 | A0A218XH64 | Acyl carrier protein | 346285.33 | 291068.67 | 438519 | 41.25 | 1924610 | 125.16 | 2.22×10^-7^ | acyl carrier protein 1, mitochondrial |
| 301 | A0A218XVI0 | Uncharacterized protein LOC116215156 | 416399 | 41.25 | 206136 | 41.25 | 7132.27 | 125.05 | 2.22×10^-7^ | uncharacterized protein LOC116215156 |
| 302 | A0A218WTN7 | Nuclear transport factor 2-like | 1147286.67 | 41.25 | 875506 | 41.25 | 605828 | 124.87 | 2.23×10^-7^ | nuclear transport factor 2-like |
| 303 | A0A218W8J9 | Cupin typ× 101 domain-containing protein | 1549386.67 | 7291340 | 13192333.33 | 23098500 | 9171986.67 | 124.66 | 2.24×10^-7^ | hypothetical protein CDL15-Pgr025060 |
| 304 | A0A218XH01 | Eukaryotic translation initiation factor 3 subunit K | 214418.67 | 41.25 | 41.25 | 41.25 | 566127.33 | 123.79 | 2.31×10^-7^ | eukaryotic translation initiation factor 3 subunit K |
| 305 | A0A6P8BMW6 | Cytochrome b-c1 complex subunit 7 | 229023.33 | 226184.33 | 379344.67 | 297695 | 925989.67 | 122.66 | 2.40×10^-7^ | cytochrome b-c1 complex subunit 7-2-like |
| 306 | A0A2I0LDJ2 | acid phosphatase | 55524.07 | 41.25 | 41.25 | 41.25 | 171261 | 122.47 | 2.41×10^-7^ | hypothetical protein CRG98-000810 |
| 307 | A0A2I0HBE2 | Cysteine proteinase inhibitor (Fragment) | 6977106.67 | 3340026.67 | 3391453.33 | 41.25 | 41498333.33 | 122.25 | 2.43×10^-7^ | hypothetical protein CRG98-049591, partial |
| 308 | A0A2I0KYG9 | Uncharacterized protein LOC116205940 | 41.25 | 41.25 | 41.25 | 41.25 | 1812.81 | 121.96 | 2.45×10^-7^ | uncharacterized protein LOC116205940 |
| 309 | A0A218WAR0 | RuBisCO large subunit-binding protein subunit beta, chloroplastic | 268862 | 155394 | 228767.33 | 272392 | 745174 | 121.86 | 2.45×10^-7^ | ruBisCO large subunit-binding protein subunit beta, chloroplastic |
| 310 | A0A218W774 | BRO1 domain-containing protein | 162204 | 41.25 | 184765.67 | 41.25 | 238883 | 121.57 | 2.47×10^-7^ | hypothetical protein CDL15-Pgr017629 |
| 311 | A0A218WZR9 | Serin× 10threonine kinase receptor-associated protein | 36278.97 | 41.25 | 57480.47 | 41.25 | 102725.67 | 121.36 | 2.47×10^-7^ | serine-threonine kinase receptor-associated protein-like |
| 312 | A0A218X2A1 | HIT domain-containing protein | 410-9380 | 10-90016.67 | 1074057.33 | 488707.67 | 6730966.67 | 121.34 | 2.47×10^-7^ | hypothetical protein CDL15-Pgr003030 |
| 313 | A0A218XQ39 | Uncharacterized protein | 141532.33 | 41.25 | 41.25 | 41.25 | 74303.43 | 121.39 | 2.47×10^-7^ | hypothetical protein CDL15-Pgr015825 |
| 314 | A0A218XTP8 | Bax inhibitor 1 | 41.25 | 41.25 | 41.25 | 41.25 | 31948.17 | 120.88 | 2.51×10^-7^ | bax inhibitor 1 |
| 315 | A0A218XL70 | Transcription factor GTE2-like | 1785.43 | 41.25 | 41.25 | 41.25 | 1189.7 | 120.66 | 2.52×10^-7^ | transcription factor GTE2-like |
| 316 | A0A2I0HL80 | TOM1-like protein 6 isoform X1 | 5752.09 | 41.25 | 41.25 | 41.25 | 3616.83 | 120.21 | 2.55×10^-7^ | TOM1-like protein 6 isoform X1 |
| 317 | A0A2I0K477 | Uncharacterized protein LOC116204645 | 98505.37 | 7631 | 63708.57 | 264889.67 | 78674.53 | 120.26 | 2.55×10^-7^ | uncharacterized protein LOC116204645 |
| 318 | A0A218WT14 | Protein phosphatase inhibitor 2-like | 494098.67 | 41.25 | 386187 | 41.25 | 644994 | 118.89 | 2.63×10^-7^ | hypothetical protein CDL15-Pgr021793 |
| 319 | A0A218X691 | Transportin-1 | 84940.9 | 41.25 | 41.25 | 41.25 | 159150.33 | 119.21 | 2.63×10^-7^ | transportin-1 |
| 320 | A0A218XLE9 | Haloacid dehalogenas× 10like hydrolase domain-containing protein 3 | 206260 | 41.25 | 93050.6 | 41.25 | 79053.1 | 119.17 | 2.63×10^-7^ | hypothetical protein CDL15-Pgr029147 |
| 321 | A0A218XS03 | Bifunctional inhibitor/plant lipid transfer protein/seed storage helical domain-containing protein | 3077330 | 11859166.67 | 6138390 | 380454 | 565206.67 | 119.1 | 2.63×10^-7^ | hypothetical protein CDL15-Pgr000382 |
| 322 | A0A218XZN9 | MA3 DOMAIN-CONTAINING TRANSLATION REGULATORY FACTOR 1-like | 388158 | 329654 | 367281.67 | 341619.67 | 1214783.33 | 119.02 | 2.63×10^-7^ | MA3 DOMAIN-CONTAINING TRANSLATION REGULATORY FACTOR 1-like |
| 323 | A0A2I0HMZ4 | Isocitrate dehydrogenase [NAD] regulatory subunit 1, mitochondrial isoform X1 | 126192.67 | 41.25 | 41.25 | 41.25 | 171101 | 119.24 | 2.63×10^-7^ | isocitrate dehydrogenase [NAD] regulatory subunit 1, mitochondrial isoform X1 |
| 324 | A0A2I0ITP1 | Zinc finger protein ZPR1-like | 312523 | 41.25 | 122931 | 41.25 | 314428.33 | 118.96 | 2.63×10^-7^ | zinc finger protein ZPR1-like |
| 325 | A0A6P8BQB8 | Vacuolar proton pump subunit B | 222614.33 | 332145.67 | 447343.33 | 541901 | 1513680 | 118.99 | 2.63×10^-7^ | V-type proton ATPase subunit B2 |
| 326 | A0A218VTL2 | 17.3 kDa class II heat shock protein-like | 17785433.33 | 6756043.33 | 27665666.67 | 3175116.67 | 17881100 | 118.59 | 2.65×10^-7^ | 17.3 kDa class II heat shock protein-like |
| 327 | A0A2I0JD82 | Uncharacterized protein | 330371.67 | 82069.73 | 195265.67 | 41.25 | 479074.67 | 118.45 | 2.66×10^-7^ | hypothetical protein CRG98-025420 |
| 328 | A0A218Y1W0 | Oleosin | 1868283.33 | 2227820 | 4561443.33 | 1000534.33 | 17541366.67 | 118.07 | 2.69×10^-7^ | hypothetical protein CDL15-Pgr014825 |
| 329 | A0A2I0KS36 | Uncharacterized protein LOC116199783 | 40976266.67 | 2615960 | 50025500 | 41.25 | 989103.67 | 118.12 | 2.69×10^-7^ | uncharacterized protein LOC116199783 |
| 330 | A0A218VWE5 | CCR4-NOT transcription complex subunit 1 | 22475.67 | 41.25 | 41.25 | 41.25 | 37069.53 | 117.89 | 2.70×10^-7^ | hypothetical protein CDL15-Pgr028614 |
| 331 | A0A218X6Q5 | Probable amidase At4g34880 | 73461.93 | 127224 | 41.25 | 41.25 | 102729.03 | 117.78 | 2.70×10^-7^ | probable amidase At4g34880 |
| 332 | A0A2I0KGK5 | Aspartate aminotransferase | 48470.97 | 41.25 | 38973.03 | 86260.27 | 36513.2 | 117.57 | 2.72×10^-7^ | aspartate aminotransferase, cytoplasmic-like |
| 333 | A0A218XNN7 | AB hydrolas× 101 domain-containing protein | 9377.68 | 41.25 | 41.25 | 41.25 | 10215.71 | 116.78 | 2.80×10^-7^ | hypothetical protein CDL15-Pgr011113 |
| 334 | A0A2I0J3W5 | Protein WEAK CHLOROPLAST MOVEMENT UNDER BLUE LIGHT 1-like | 44969.2 | 41.25 | 54781.73 | 41.25 | 72173.97 | 116.53 | 2.82×10^-7^ | protein WEAK CHLOROPLAST MOVEMENT UNDER BLUE LIGHT 1-like |
| 335 | A0A6P8CX91 | Calnexin homolog | 635634.67 | 687779 | 786768 | 782933.33 | 2126933.33 | 115.8 | 2.90×10^-7^ | calnexin homolog |
| 336 | A0A218WLY8 | Methyl-CpG-binding domain-containing protein 11-like isoform X2 | 134635.33 | 41.25 | 74885.3 | 41.25 | 71806.37 | 115.52 | 2.92×10^-7^ | methyl-CpG-binding domain-containing protein 11-like isoform X2 |
| 337 | A0A218XEM3 | Tubulin-specific chaperone A | 1193703.33 | 78571.5 | 1477573.33 | 41.25 | 553198.67 | 115.4 | 2.93×10^-7^ | tubulin-folding cofactor A |
| 338 | A0A2I0L2M6 | NifU-like protein 2, chloroplastic isoform X1 | 46124.4 | 41.25 | 41.25 | 41.25 | 128179.67 | 115.1 | 2.96×10^-7^ | nifU-like protein 2, chloroplastic isoform X1 |
| 339 | A0A6P8DNE6 | ESCRT-II complex subunit VPS25 | 50727.47 | 41.25 | 4117.33 | 41.25 | 92221.63 | 114.92 | 2.97×10^-7^ | vacuolar protein sorting-associated protein 25 |
| 340 | A0A218XRS2 | RNA-binding KH domain-containing protein PEPPER-like | 27356.93 | 41.25 | 41.25 | 41.25 | 65546.8 | 114.78 | 2.98×10^-7^ | RNA-binding KH domain-containing protein PEPPER-like |
| 341 | A0A218WCR5 | DNA 3'-5' helicase | 118998.73 | 240255.33 | 10-9476.42 | 41.25 | 1012913.33 | 114.32 | 3.03×10^-7^ | hypothetical protein CDL15-Pgr014317 |
| 342 | A0A218WI98 | Reticulon-like protein | 1366453.33 | 1383805 | 10-90026.67 | 1400055 | 7684423.33 | 114.27 | 3.03×10^-7^ | reticulon-like protein B1 |
| 343 | A0A218XGT9 | Glycin× 10rich protein 2-like | 3372540 | 3689213.33 | 4717380 | 515039.67 | 7204406.67 | 113.98 | 3.06×10^-7^ | glycine-rich protein 2-like |
| 344 | A0A218XFL3 | Oil body-associated protein 1A-like | 5967880 | 1205635.33 | 1367333.33 | 938959.33 | 3034780 | 113.54 | 3.10×10^-7^ | oil body-associated protein 1A-like |
| 345 | A0A6P8CYV7 | Short-chain dehydrogenase TIC 32, chloroplastic-like | 83460.67 | 41.25 | 94609.23 | 41.25 | 63626.97 | 113.49 | 3.10×10^-7^ | short-chain dehydrogenase TIC 32, chloroplastic-like |
| 346 | A0A218WVI2 | Uncharacterized protein | 56176.2 | 41.25 | 41.25 | 41.25 | 185821.33 | 113.28 | 3.11×10^-7^ | hypothetical protein CDL15-Pgr009637 |
| 347 | A0A218XED2 | Ultraviolet-B receptor UVR8 | 60364.87 | 214408.67 | 41.25 | 41.25 | 80979.1 | 113.32 | 3.11×10^-7^ | hypothetical protein CDL15-Pgr029202 |
| 348 | A0A218VZ66 | Transcription factor CBF/NF-Y/archaeal histone domain-containing protein | 32908.17 | 41.25 | 41.25 | 41.25 | 57948 | 113.21 | 3.12×10^-7^ | hypothetical protein CDL15-Pgr008759 |
| 349 | A0A218VYN3 | Protein HEAT INTOLERANT 4-like | 47119 | 41.25 | 89272.4 | 41.25 | 129343 | 112.96 | 3.14×10^-7^ | protein HEAT INTOLERANT 4-like |
| 350 | A0A218W936 | NADP-dependent oxidoreductase domain-containing protein | 132766.33 | 41.25 | 41.25 | 41.25 | 194114.67 | 112.79 | 3.15×10^-7^ | hypothetical protein CDL15-Pgr025175 |
| 351 | A0A218XCC6 | Uncharacterized protein | 46031.73 | 41.25 | 41.25 | 41.25 | 117697 | 112.36 | 3.21×10^-7^ | hypothetical protein CDL15-Pgr002168 |
| 352 | A0A218XUR8 | Histone H1 | 3246.36 | 41.25 | 41.25 | 41.25 | 3509.61 | 111.86 | 3.27×10^-7^ | histone H1 |
| 353 | A0A218WLQ0 | Membrin | 211421.33 | 41.25 | 191840 | 41.25 | 213386 | 111.77 | 3.27×10^-7^ | hypothetical protein CDL15-Pgr001295 |
| 354 | A0A218W7G1 | 17.4 kDa class III heat shock protein | 2006916.67 | 359740.33 | 606701.67 | 41.25 | 1637136.67 | 111.66 | 3.28×10^-7^ | 17.4 kDa class III heat shock protein |
| 355 | A0A218WD74 | Uncharacterized protein At2g39795, mitochondrial-like | 918472.67 | 2928523.33 | 639217.67 | 41.25 | 2288976.67 | 111.55 | 3.28×10^-7^ | uncharacterized protein At2g39795, mitochondrial-like |
| 356 | A0A218WIT4 | Mitochondrial proton/calcium exchanger protein | 924384.67 | 69240.17 | 495469 | 41.25 | 2223526.67 | 111.37 | 3.30×10^-7^ | mitochondrial proton/calcium exchanger protein |
| 357 | A0A218VXY0 | Amine oxidase | 793.32 | 41.25 | 41.25 | 6629.26 | 832.85 | 111.11 | 3.32×10^-7^ | uncharacterized protein LOC116212918 |
| 358 | A0A218XVW6 | Electron transfer flavoprotein subunit alpha | 84137.27 | 41.25 | 41.25 | 41.25 | 53112.23 | 111.07 | 3.32×10^-7^ | electron transfer flavoprotein subunit alpha, mitochondrial |
| 359 | A0A218WHZ8 | Peptidase A1 domain-containing protein | 12048.8 | 41.25 | 41.25 | 41.25 | 19169.07 | 110.57 | 3.38×10^-7^ | hypothetical protein CDL15-Pgr018191 |
| 360 | A0A218X3Z9 | RRM domain-containing protein | 31775.27 | 41.25 | 41.25 | 41.25 | 26632.4 | 110.59 | 3.38×10^-7^ | hypothetical protein CDL15-Pgr022828 |
| 361 | A0A218X8N8 | Glutathione S-transferase | 5054563.33 | 618233.67 | 551825.67 | 1785816.67 | 1679863.33 | 10-9.66 | 3.51×10^-7^ | probable glutathione S-transferase |
| 362 | A0A218W2B1 | Mitochondrial amidoxim× 10reducing component 1-like | 24059.9 | 41.25 | 41.25 | 41.25 | 41.25 | 10-9.43 | 3.53×10^-7^ | mitochondrial amidoxime-reducing component 1-like |
| 363 | A0A218VYS9 | Cell division cycle protein 48 homolog | 147699.33 | 146551.33 | 254609 | 681886.67 | 524162 | 10-9.23 | 3.55×10^-7^ | cell division cycle protein 48 homolog |
| 364 | A0A218WCK6 | Uncharacterized protein | 18319.87 | 41.25 | 41.25 | 41.25 | 16422.73 | 10-9.17 | 3.55×10^-7^ | hypothetical protein CDL15-Pgr026113 |
| 365 | A0A218WY02 | HBS1-like protein | 1069.77 | 41.25 | 2008.97 | 41.25 | 41.25 | 10-9.18 | 3.55×10^-7^ | hypothetical protein CDL15-Pgr028897 |
| 366 | A0A218Y2D4 | Protein THYLAKOID FORMATION1, chloroplastic | 41.25 | 41.25 | 41.25 | 41.25 | 110-936.6 | 10-9.03 | 3.56×10^-7^ | protein THYLAKOID FORMATION1, chloroplastic |
| 367 | A0A2I0ILG7 | T-complex protein 1 subunit alpha | 169772.67 | 41.25 | 254444.67 | 705356.33 | 407527 | 108.97 | 3.56×10^-7^ | T-complex protein 1 subunit alpha |
| 368 | A0A218WPA1 | 3-methyl-2-oxobutanoate hydroxymethyltransferase | 98919.2 | 41.25 | 41.25 | 41.25 | 53643.07 | 108.13 | 3.68×10^-7^ | 3-methyl-2-oxobutanoate hydroxymethyltransferase 1, mitochondrial-like |
| 369 | A0A218Y287 | Major latex allergen Hev b 5 | 579595 | 182487 | 359353 | 41.25 | 376005 | 108.09 | 3.68×10^-7^ | major latex allergen Hev b 5 |
| 370 | A0A6P8CDH8 | Uncharacterized protein LOC116195823 | 4522500 | 1381740 | 7869196.67 | 41.25 | 383435 | 107.39 | 3.79×10^-7^ | uncharacterized protein LOC116195823 |
| 371 | A0A218XN02 | Acyl carrier protein | 213851 | 41.25 | 223012.08 | 41.25 | 1776663.33 | 107.24 | 3.81×10^-7^ | acyl carrier protein 2, mitochondrial |
| 372 | A0A218VVL2 | Non-specific phospholipase C3-like | 41.25 | 41.25 | 41.25 | 41.25 | 8479.06 | 106.92 | 3.85×10^-7^ | hypothetical protein CDL15-Pgr014099 |
| 373 | A0A218VWA3 | Late embryogenesis abundant protein Lea14-A-like | 8382913.33 | 1142605 | 2460240 | 427240.83 | 5090523.33 | 106.75 | 3.87×10^-7^ | late embryogenesis abundant protein Lea14-A-like |
| 374 | A0A218W522 | Uncharacterized protein | 7241.48 | 41.25 | 41.25 | 41.25 | 13505.93 | 106.44 | 3.90×10^-7^ | hypothetical protein CDL15-Pgr028431 |
| 375 | A0A218W7W4 | ATP-dependent Clp protease proteolytic subunit | 291290.67 | 41.25 | 240050 | 41.25 | 327734.67 | 106.48 | 3.90×10^-7^ | ATP-dependent Clp protease proteolytic subunit 4, chloroplastic |
| 376 | A0A2I0IYQ8 | Peptidase A1 domain-containing protein | 1384996.67 | 4102360 | 16276866.67 | 1798963.33 | 7374220 | 106.23 | 3.93×10^-7^ | hypothetical protein CRG98-030721 |
| 377 | A0A218Y2P6 | UspA domain-containing protein | 1443523.33 | 274106 | 942657 | 567033.33 | 2552243.33 | 106.07 | 3.95×10^-7^ | hypothetical protein CDL15-Pgr000272 |
| 378 | A0A218VQL7 | Nitrogen regulatory protein P-II homolog isoform X2 | 6955830 | 27413833.33 | 6583576.67 | 1853176.67 | 9111760 | 105.8 | 3.99×10^-7^ | nitrogen regulatory protein P-II homolog isoform X2 |
| 379 | A0A218XFJ4 | Eukaryotic translation initiation factor 4G | 78698.73 | 41.25 | 71551.67 | 41.25 | 43659.77 | 105.55 | 4.02×10^-7^ | hypothetical protein CDL15-Pgr004123 |
| 380 | A0A6P8CC04 | Regulator of nonsense transcripts UPF2 | 24023.57 | 41.25 | 41.25 | 41.25 | 40122 | 105.07 | 4.10×10^-7^ | regulator of nonsense transcripts UPF2 |
| 381 | A0A218XN66 | RRM domain-containing protein | 353970.67 | 41.25 | 41.25 | 41.25 | 340355 | 104.51 | 4.19×10^-7^ | hypothetical protein CDL15-Pgr010696 |
| 382 | A0A6P8CGJ6 | Elongation factor 1-gamma-like | 2059690 | 390047.67 | 1198003.33 | 951464.33 | 441075.67 | 104.53 | 4.19×10^-7^ | elongation factor 1-gamma-like |
| 383 | A0A218VSA8 | Lon protease homolog, mitochondrial | 70725.83 | 41.25 | 66416.83 | 41.25 | 61756.83 | 104.34 | 4.21×10^-7^ | lon protease homolog 1, mitochondrial-like isoform X1 |
| 384 | A0A6P8E9L8 | phenylalanin× 10-tRNA ligase | 60980.33 | 41.25 | 85655 | 41.25 | 76096.77 | 104.04 | 4.26×10^-7^ | phenylalanine--tRNA ligase beta subunit, cytoplasmic |
| 385 | A0A218W432 | low-specificity L-threonine aldolase | 41.25 | 41.25 | 41.25 | 41.25 | 270917.67 | 103.69 | 4.32×10^-7^ | hypothetical protein CDL15-Pgr000755 |
| 386 | A0A218VTT0 | Ubiquinol oxidase | 41.25 | 41.25 | 41.25 | 41.25 | 35606.6 | 103.44 | 4.36×10^-7^ | ubiquinol oxidase, mitochondrial |
| 387 | A0A218WG60 | Glutaredoxin domain-containing protein | 1214670 | 510578.33 | 576272.33 | 106889.75 | 1736956.67 | 103.31 | 4.37×10^-7^ | hypothetical protein CDL15-Pgr005710 |
| 388 | A0A2I0J659 | 26S proteasome non-ATPase regulatory subunit 8 homolog A | 700319 | 136987.75 | 222637 | 41.25 | 1228456.67 | 103.24 | 4.38×10^-7^ | 26S proteasome non-ATPase regulatory subunit 8 homolog A |
| 389 | A0A6P8CEC7 | Fimbrin-5 | 810316 | 41.25 | 979799.67 | 41.25 | 421919.33 | 103.02 | 4.41×10^-7^ | fimbrin-5 |
| 390 | A0A2I0K1N3 | Pterin-binding domain-containing protein | 41.25 | 41.25 | 41.25 | 41.25 | 125439 | 102.96 | 4.41×10^-7^ | hypothetical protein CRG98-017086 |
| 391 | A0A218W474 | Uncharacterized protein | 21957.4 | 41.25 | 41.25 | 41.25 | 21498.3 | 102.18 | 4.57×10^-7^ | hypothetical protein CDL15-Pgr024749 |
| 392 | A0A218X6F4 | Uncharacterized protein LOC116194082 | 97123.5 | 41.25 | 19247.63 | 41.25 | 291548.67 | 101.32 | 4.75×10^-7^ | uncharacterized protein LOC101255006 |
| 393 | A0A218Y016 | Probable calcium-binding protein CML29 | 1180.93 | 41.25 | 41.25 | 41.25 | 2452.39 | 101.26 | 4.75×10^-7^ | probable calcium-binding protein CML29 |
| 394 | A0A218Y1J8 | Profilin | 10247150 | 4780520 | 5566533.33 | 1410760 | 5305946.67 | 100.88 | 4.82×10^-7^ | profilin-1 |
| 395 | A0A218W4V7 | RING-type E3 ubiquitin transferase | 23025.13 | 41.25 | 41.25 | 41.25 | 41.25 | 100.75 | 4.84×10^-7^ | vacuolar protein sorting-associated protein 52 A |

**Table S2.**

Secondary structure composition of different PSP fractions

| Protein sample | α-Helix (%) | β-Sheet (%) | β-Turn (%) | Random coil (%) |
| --- | --- | --- | --- | --- |
| ALB | 33.14 ± 1.09^c^ | 38.53 ± 1.23^a^ | 12.76 ± 1.24^bcd^ | 16.49 ± 1.30^acd^ |
| GLO | 39.05 ± 0.21^b^ | 31.67 ± 1.06^cd^ | 16.40 ± 1.02^a^ | 12.95 ± 1.90^bce^ |
| PRO | 43.84 ± 1.25^a^ | 30.12 ± 1.18^ce^ | 12.43 ± 1.52^be^ | 14.05 ± 1.72^aef^ |
| GLU | 36.98 ± 1.34^bd^ | 34.99 ± 0.74^b^ | 16.30 ± 1.35^ac^ | 11.68 ± 1.84^bdf^ |
| CP | 34.91 ± 1.03^cd^ | 32.14 ± 1.36^bde^ | 15.14 ± 1.46^ade^ | 18.01 ± 2.13^a^ |

Values are presented as mean ± standard deviation (n = 3). Different lowercase superscript letters indicate significant differences among samples (*P < 0.05*).

**Table S3.**

Protein purity and protein extraction recovery of different PSP fractions

| Protein fraction | Protein purity (%, wet basis) | Protein extraction recovery (%) |
| --- | --- | --- |
| CP | 63.77 ± 1.89 | 64.62 |
| ALB | 83.58 ± 2.32 | 19.38 |
| GLO | 54.40 ± 0.93 | 46.42 |
| PRO | 69.95 ± 0.93 | 7.63 |
| GLU | 60.21 ± 0.14 | 24.18 |

**Table S4.**

High-confidence potential allergenic proteins identified of PSP different fractions.

| Accession | Allergenic protein | Protein annotation | ALB | GLO | PRO | GLU | CP | Evidence basis | Reference |
| --- | --- | --- | --- | --- | --- | --- | --- | --- | --- |
| A0A059STC4 | Pun g 1.0101 | Non-specific lipid-transfer protein (nsLTP) | 21.67 ± 2.87 | 93.37 ± 9.21 | 108.2 ± 21.6 | 0.601 ± 0.236 | 1.178 ± 0.067 | Exact accession corresponds to a pomegranate Pun g 1 isoallergen; IgE-binding and cross-reactivity were experimentally demonstrated. | O’Malley et al., 2021 |
| A0A059SSZ0 | Pun g 1.0201 | Non-specific lipid-transfer protein (nsLTP) | 1.914 ± 0.098 | 19.86 ± 5.72 | 15.12 ± 3.20 | 0.829 ± 0.181 | 0.270 ± 0.057 | Exact accession corresponds to a pomegranate Pun g 1 isoallergen; IgE-binding and cross-reactivity were experimentally demonstrated. | O’Malley et al., 2021 |
| A0A059ST23 | Pun g 1.0301 | Non-specific lipid-transfer protein (nsLTP) | 3.077 ± 0.438 | 17.79 ± 2.54 | 14.43 ± 2.40 | 0.168 ± 0.000 (n=2) | 0.391 ± 0.033 | Exact accession corresponds to a pomegranate Pun g 1 isoallergen; IgE-binding and cross-reactivity were experimentally demonstrated. | O’Malley et al., 2021 |
| G1UH28 | Pun g 14 | Acidic endochitinase / chitinase III | 42.36 ± 6.30 | 30.54 ± 8.22 | 82.86 ± 19.02 | 11.50 ± 4.13 | 2.534 ± 0.133 | WHO/IUIS-recognized pomegranate food allergen; IgE binding was demonstrated by dot blot, immunoblotting, and FABER testing. | Tuppo et al. (2018) |
| A0A218XU94 | Pun g 2S-A1 | 2S albumin | 2105.1 ± 175.5 | 6364.4 ± 2132.0 | 5610.6 ± 1459.5 | 496.3 ± 130.6 | 1818.2 ± 58.9 | Exact accession of a pomegranate seed 2S albumin isolated and characterized experimentally; specific IgE binding was demonstrated. | Tuppo et al. (2024) |
| A0A2I0JHZ1 | Pun g 2S-A2 | 2S albumin | 1466.0 ± 296.8 | 4034.3 ± 1159.3 | 6027.6 ± 1381.7 | 127.3 ± 34.1 | 1142.8 ± 164.7 | Exact accession of a pomegranate seed 2S albumin isolated and characterized experimentally; specific IgE binding was demonstrated. | Tuppo et al. (2024) |

Note: Values are expressed as mean ± SD of relative DIA-MS abundance (×10⁶, a.u.). *Pun g 1.0301 in GLU was detected in two of three replicates. Proteins were selected based on reported IgE-binding evidence; proteomic detection does not confirm clinical allergenicity.
